# New *Campylobacter* Lineages in New Zealand Freshwater: Pathogenesis and Public Health Implications

**DOI:** 10.1111/1462-2920.70016

**Published:** 2024-12-16

**Authors:** Adrian L. Cookson, Sara Burgess, Anne C. Midwinter, Jonathan C. Marshall, Marie Moinet, Lynn Rogers, Ahmed Fayaz, Patrick J. Biggs, Gale Brightwell

**Affiliations:** ^1^ AgResearch Limited Hopkirk Research Institute Palmerston North New Zealand; ^2^ mEpiLab, School of Veterinary Sciences Massey University Palmerston North New Zealand; ^3^ School of Mathematical and Computational Sciences Massey University Palmerston North New Zealand; ^4^ School of Natural Sciences Massey University Palmerston North New Zealand

**Keywords:** *Campylobacter coli*, *Campylobacter jejuni*, land‐use, naturalised, pathogen, persistent, water quality

## Abstract

This study investigated the diversity of thermophilic *Campylobacter* species isolated from three New Zealand freshwater catchments affected by pastoral and urban activities. Utilising matrix‐assisted laser desorption ionisation‐time of flight and whole genome sequence analysis, the study identified *Campylobacter jejuni* (*n* = 46, 46.0%), *C*. *coli* (*n* = 39, 39%), *C*. *lari* (*n* = 4, 4.0%), and two novel *Campylobacter* species lineages (*n* = 11, 11%). Core genome sequence analysis provided evidence of prolonged persistence or continuous faecal shedding of closely related strains. The *C*. *jejuni* isolates displayed distinct sequence types (STs) associated with human, ruminant, and environmental sources, whereas the *C*. *coli* STs included waterborne ST3302 and ST7774. Recombination events affecting loci implicated in human pathogenesis and environmental persistence were observed, particularly in the cdtABC operon (encoding the cytolethal distending toxin) of non‐human *C*. *jejuni* STs. A low diversity of antimicrobial resistance genes (aadE‐Cc in *C*. *coli* ), with genotype/phenotype concordance for tetracycline resistance (tetO) in three ST177 isolates, was noted. The data suggest the existence of two types of naturalised waterborne *Campylobacter*: environmentally persistent strains originating from waterbirds and new environmental species not linked to human campylobacteriosis. Identifying and understanding naturalised *Campylobacter* species is crucial for accurate waterborne public health risk assessments and the effective allocation of resources for water quality management.

## Graphical abstract

The analysis of whole genome sequence data has identified freshwater *Campylobacter*, providing insights into pathogen diversity, antimicrobial resistance, and recombination events affecting virulence. This distinguishes human‐linked strains from wildlife‐origin strains, findings important for refining waterborne public health risk assessments and water quality management.

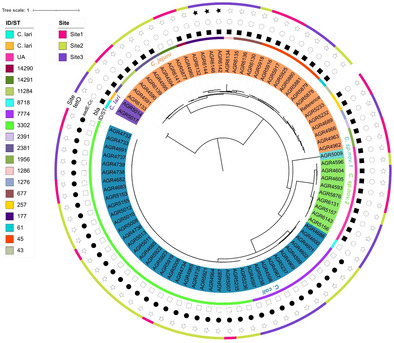

## 1 Introduction

Thermophilic *Campylobacter* species are the leading cause of bacterial gastroenteritis in humans worldwide (Anon 2020a). Poultry, ruminants, and to a lesser extent wildlife, are the main reservoirs of infection (Lake et al. 2021; Phiri et al. 2020a; Shrestha et al. 2019) and environmental contamination with faecal material has also been associated with large outbreaks of human disease (Gilpin et al. 2020). Surveillance reports from 2022 describe an overall European Union/European Economic Area notification rate of 46.9 cases per 100,000 population (Anon 2024a), but in New Zealand where there were 5729 cases of campylobacteriosis notified in 2021, the rate was 111.8 per 100,000 with an estimated 75% associated with foodborne transmission (Anon 2024b). In New Zealand, campylobacteriosis is the most commonly notified disease accounting for 47.3% of all notifications (excluding COVID‐19 notifications) in 2021 (Anon 2024b). Data from a New Zealand case–control study to determine source attribution of human campylobacteriosis cases demonstrated that most cases (84%) were infected with strains attributed to a poultry source and 14% were attributed to a cattle source (Lake et al. 2021), but factors involved in pathogen transmission to humans was not examined. Contact with farm animals (43.8%) and consumption of food from retail premises (43.5%) were the most common risk factors reported for campylobacteriosis followed by consumption of untreated water (29.4%) and recreational water contact (11.3%) (Anon 2024b).

Water contaminated with faecal material can harbour a variety of human pathogens such as *Campylobacter*, Shiga toxin‐producing *Escherichia coli* , *Salmonella* and protozoa including *Cryptosporidium* and *Giardia* that may cause human disease (Cookson et al. 2024; Phiri et al. 2020b; Hörman et al. 2004). However, conventional water quality analyses to assess faecal contamination and estimate human health risk rely on the enumeration of *E*. *coli* , as it is relatively easy and cost effective to culture and detect. Unlike several other waterborne pathogens, *E*. *coli* are able to grow quickly and allow for rapid responses to potential outbreaks of waterborne disease to protect public health. Thus, *E*. *coli* is used as a faecal indicator bacterium and when present at high concentrations implies an increased risk of other slower growing waterborne pathogens, such as *Campylobacter* (Till et al. 2008).

Phylogenetic analyses have assisted with the typing and source attribution of waterborne pathogens to provide improved human health risk assessments (Lake et al. 2021; Liao et al. 2019; Harrison et al. 2021). For example, high resolution genomics has demonstrated the presence of two types of naturalised populations of faecal indicator bacteria; *E*. *coli* from non‐recent faecal contamination events able to survive and persist in the environment (Luo et al. 2011; Cookson et al. 2022; Nowicki et al. 2021), and ‘cryptic’ non‐ *E*. *coli* *Escherichia* species, phenotypically indistinguishable from *E*. *coli* , but distinct at the genomic level, that are rarely isolated from human or ruminant faecal samples (Walk 2015). Furthermore, analysis of *E*. *coli* populations from freshwater samples collected at sites impacted by different land‐uses including urban, pastoral or low impact/native forest have demonstrated that the newly described *Escherichia* species are generally found in low concentrations and are therefore unlikely to confound water quality assessments (Cookson et al. 2024).

Analogous to the identification of the ‘cryptic’ *Escherichia* species (Walk 2015; Koh et al. 2022) and their uncertain role as indicators of faecal contamination, certain thermophilic *Campylobacter* STs, absent from human clinical cases, can be widely isolated from wildlife and environmental sources (Phiri et al. 2020a; Shrestha et al. 2019; Carter et al. 2009; Nohra et al. 2016; Irshad et al. 2016; Inglis, Teixeira, and Boras 2021). Additionally, exemplar environmental STs such as *C*. *jejuni* ST2381, and *C*. *coli* ST3302 and ST7774 appear geographically confined having been previously isolated only from New Zealand (Jolley, Bray, and Maiden 2018). The geospatial isolation of certain thermophilic *Campylobacter* lineages suggests not only the isolated evolution of localised STs, but that their apparent lack of pathogenicity in human disease is associated with a specialised wildlife host source and transmission to the environment.

Genetic variation of thermophilic *Campylobacter* is mediated through mutation and recombination (Biggs et al. 2011) and is thought to facilitate niche specialisation and host adaptation (Sheppard et al. 2013; Mourkas et al. 2021). Increased efficiency of adaptation occurs in lineages through combinations of beneficial alleles fixed by selection and enhanced recombination (Woodcock et al. 2017). However, for generalist *Campylobacter* STs, such as ST45, which are able to colonise multiple‐hosts (Dearlove et al. 2016; Méric et al. 2018), lower levels of recombination occur increasing population variation allowing a more effective response to changes in the selective landscape (Woodcock et al. 2017). Specifically, specialisation promoted by recombination allows a more rapid response after a genetic bottleneck, but is a potentially precarious strategy as environmental changes may cause extinction if specialist STs, such as 2381 and 2391, are unable to move between niches/hosts (Woodcock et al. 2017). For those generalist STs likely to encounter frequent host switches, a lower recombination rate may be optimal. Thus, the transfer of ecologically important loci amongst co‐existing *Campylobacter* strains contributes to the host specificity and generalism observed in parallel with geographically localised populations (Biggs et al. 2011; Sheppard et al. 2013; Mourkas et al. 2021; Poorrashidi, Hitchcock, and Xu 2024).

Accurate microbial freshwater quality assessments for recreational water contact are important to assess public health risk and prioritise investigations by water managers which identify faecal sources and mitigation strategies to reduce pathogen contamination (Till et al. 2008). In this work, we describe detailed land‐use categories from three freshwater catchments and the analysis of thermophilic *Campylobacter* species obtained from surface water samples. This current study utilises a genomic epidemiology approach to undertake risk profiling of isolates using conventional multilocus sequence typing (MLST), core genome single nucleotide polymorphism (SNP) analysis, and the putative identification of novel thermophilic *Campylobacter* lineages connected to environmental sites.

## 2 Material and Methods

**FIGURE 1:**
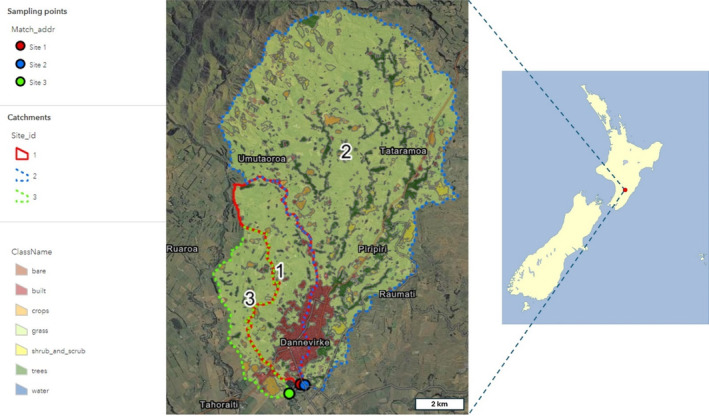
Sampling sites included in this study. Site 1, Tapuata (red); Site 2, Mangatera (blue); and Site 3, Mākirikiri (green). Land cover data were established from the Land Cover Database (v. 5.0) with visualisation using Dynamic World Classified Imagery for the March 2020—March 2021 sampling period.

Field sites for this study are comprised of three separate freshwater catchments as part of the wider Eastern Manawatū River in the lower North Island, New Zealand, close to Dannevirke in the Tararua region (Figure 1). The Tapuata Stream (Site 1, Latitude: −40.223700; Longitude: 176.099697) has a mixed urban and pastoral (ruminant livestock farming) observed land‐use; the Mangatera Stream (Site 2, Latitude: −40.224467; Longitude: 176.10157540) has a pastoral (ruminant livestock farming) observed land‐use; and the Mākirikiri Stream (Site 3, Latitude: −40.227225; Longitude: 176.094264) also has a pastoral (ruminant livestock farming) observed land‐use (Table S1). All three sample sites are adjacent geographically; Site 1 on the Tapuata Stream is approximately 200 m upstream from its confluence with the Mangatera Stream; Site 2 on the Mangatera Stream is approximately 20 m upstream of the Tapuata Stream/Mangatera Stream confluence and 300 m upstream of an authorised discharge from the Dannevirke wastewater treatment plant; and Site 3 on the Mākirikiri Stream is approximately 250 m from its confluence with the Mangatera Stream, which is itself 600 m downstream of the Tapuata Stream/Mangatera Stream confluence. Land cover data were retrieved from the Land Cover Database (v 5.0) (Anon 2021), and riverine data from the River Environment Classification 2 (REC2) (Anon 2020b) using ipyleaflet within Jupyter, and Python (v. 3.9) scripts. Total area of sites ranged from 970 to 10,073 ha with catchment lengths of between 8.4 and 24.9 km (Table S1). As a measure of total area, grassland ranged from approximately 86% to 96% for each catchment with livestock from all three catchments dominated by sheep (69.0% to 72.0%). Land‐use of Site 1 included much of the urban infrastructure of Dannevirke (10.3% of the catchment area) (Table S1).

The sites were visited on 11 occasions over a 13‐month period from March 2020 to March 2021. On each sampling occasion, surface water was collected from the mid‐point of the stream, stored chilled (< 10°C) and processed within 8 h at the Hopkirk Research Institute (Palmerston North). *E*. *coli* were enumerated (MPN per 100 mL water) using Colilert‐18/Quanti‐Tray/2000 with incubation for 18 h at 35°C. Water (500 mL) was filtered through a 0.45 μm nitrocellulose filter (S‐Pak Filters, Millipore, Merck, Burlington, MA, USA) using a bench‐top negative pressure system (Cookson et al. 2022). Filters were then incubated in 20 mL Bolton broth, consisting of *Campylobacter* Enrichment broth, CVTN (cefoperazone, vancomycin, trimethoprim, amphotericin B) selective supplement and lysed horse blood (Fort Richard, Auckland, New Zealand) (Nohra et al. 2016). The broth was incubated for 48 h at 42°C in a microaerobic atmosphere (85% N_2_, 10% CO_2_, 5% O_2_) before an aliquot was plated onto modified charcoal‐cefoperazone‐deoxycholate agar (mCCDA) agar (Fort Richard, Auckland, New Zealand) with a sterile cotton swab. mCCDA plates were examined after 48 h of incubation at 42°C in a microaerobic atmosphere. Generally, at least two presumptive *Campylobacter* isolates were sub‐cultured onto fresh mCCDA agar followed by Columbia horse blood agar (Fort Richard, Auckland, New Zealand) (Nohra et al. 2016). Fresh microbial growth from the horse blood agar was resuspended in Nutrient Broth No. 2 (Oxoid, Hampshire, UK) and glycerol (33% [w/v]) and stored at −80°C. The ‘in tube formic acid extraction’ technique (Lévesque et al. 2015) was performed on two or three colonies from horse blood agar resuspended in sterile molecular biology grade water, and then identified using matrix‐assisted laser desorption ionisation‐time of flight (MALDI‐TOF) mass spectrometry (Bruker, Billerica, CA, USA) (Lévesque et al. 2015) using the MBT Library BDAL 9.0 (Bruker Daltonics, Germany) which includes 8,468 mass spectrum profiles. Primary evaluation for antimicrobial resistance was undertaken on Mueller‐Hinton EUCAST Agar F with horse blood (5% [v/v]) and β‐NAD (20 mg/L) (Fort Richard, Auckland, New Zealand) using MASTDISCS containing ampicillin (10 μg), erythromycin (15 μg), nalidixic acid (30 μg), penicillin G (1 unit), tetracycline (30 μg), gentamicin (10 μg), and ciprofloxacin (5 μg) (Fort Richard, Auckland, New Zealand) following the methodology prescribed by EUCAST (2021) and interpreted, as previously described (French et al. 2024). The susceptible *C*. *jejuni* strain ATCC33560 was used as a control.

Genomic DNA was extracted using the Promega wizard genomic DNA purification kit as previously described (Madison, WI, USA) (Gray et al. 2021). Libraries were prepared using the Nextera XT DNA library preparation kit (Illumina Inc., San Diego, USA) and whole genome sequencing (WGS) was undertaken by Novogene Limited (Singapore) using the Illumina HiSeq paired‐end v4 platform (2 × 125 bp). The Nullarbor (v. 2.0) pipeline (Seemann et al. 2019) was used to process and examine WGS data, which included read trimming of adaptors (trimmomatic v. 0.39) (Bolger, Lohse, and Usadel 2014), *de novo* genome assembly using SKESA (v.2.3.0) (Souvorov, Agarwala, and Lipman 2018), bacterial classification using Centrifuge (v. 1.0.4) (Kim et al. 2016), annotation using Prokka (v. 1.13.3) (Seemann 2014), and phylogenetic analysis using Snippy (v. 4.3.6) (Seemann 2018) with *C*. *jejuni* NCTC11168 (accession AL111168) as the reference sequence. Regions of recombination were identified by inputting a whole genome Fasta SNP alignment generated from Snippy into Gubbins (v. 2.3.4) (Croucher et al. 2014), with outputs visualised using Phandango (v. 1.3) (Hadfield et al. 2018). Phylogenetic analysis of aligned amino acid sequences and reconstruction of phylogenetic trees was undertaken with the Maximum Likelihood algorithm (Jones, Taylor, and Thornton 1992) using MEGA11 (Tamura, Stecher, and Kumar 2021). ABRicate (v. 0.8.13) (Seemann 2020a) was used for the mass screening of virulence using Virulence Factor Database (Chen et al. 2016), and antimicrobial resistance genes with MEGARes (v. 2.0) (Doster et al. 2020) with sequence identity and alignment coverage both set to be > 80%. MLST ST (Dingle et al. 2001) of assembled contigs was performed using pubMLST (Jolley, Bray, and Maiden 2018) (MLST, v. 2.16.1) (Seemann 2020b). *Campylobacter* phylogenies were generated from core SNP alignments (with *C*. *jejuni* NCTC11168 (accession AL111168) as the reference genome) in Snippy using the Jukes‐Cantor DNA evolution model. A phylogenetic tree was inferred by maximum likelihood using IQ‐TREE (Nguyen et al. 2014) and the resultant tree imported into the Interactive Tree of Life (v. 6.0) (Letunic and Bork 2024) software as a Newick file for subsequent annotation with experiment metadata. Clinker (v. 0.0.23) (Gilchrist and Chooi 2020) was used for genome clustering and comparative analysis of regions impacted by recombination. All tools were used with default settings unless stated otherwise.

Linear mixed effects models were applied using the lmeTest (Kuznetsova, Brockhoff, and Christensen 2017) (v. 3.1–3) R package where the effects of ‘Site’ were included as the explanatory variable in a standard linear regression, including ‘Visit’ as a random effect, with the Colilert data (logMPN) from each of the water samples. In a separate analysis, presence of any association was determined between *E*. *coli* logMPN and ‘Ruminants’ (total upstream, n), ‘Area’ (catchment area, ha), and ‘Built’ (catchment area, ha). Data visualisation and statistical analysis were performed using R (v. 3.6.2) (R Development Core Team 2018).

## 3 Results

Water samples (*n* = 33) were obtained from three sites (Figure 1) during a longitudinal study to determine *E*. *coli* concentration (MPN per 100 mL) and to filter and enrich bacteria captured on the membrane filters for subculture and selective growth of *Campylobacter* species. *E*. *coli* (MPN per 100 mL) concentrations for the three sites (Site 1, min 325.5, max 1119.9, geometric mean, 637.8; Site 2, min 248.1, max 1986.3, geometric mean, 681.7; Site 3, min 42.6, max 686.7, geometric mean 183.1) suggested that when compared to Site 3, increased *E*. *coli* concentrations were associated with Site 1 (OR = 1.72, 95% CI 1.38–2.14, *p* = 0.0001) and Site 2 (OR = 1.77, 95% CI 1.42–2.21, *p* < 0.0001). There was no difference in *E*. *coli* concentrations between Sites 2 and 3 (OR = 1.03, 95% CI 0.83–1.28, *p* = 0.80). *E*. *coli* concentration (MPN per 100 mL) was positively correlated with number of ruminants in the catchments (*p* = 0.029), catchment area (*p* = 0.026) and built/urban area (*p* = 0.011).

Presumptive *Campylobacter* were isolated from most water samples (30 of 33, 90.9%); although isolates were recovered on each sampling occasion from Sites 2 and 3, on three occasions sample enrichments from Site 1 (Visits 6, 10, and 11) gave rise to heavy growth of *E*. *coli* on the selective mCCDA medium (Burgess et al. 2022) which precluded the subculture of putative *Campylobacter* isolates. In total, 100 putative *Campylobacter* strains were recovered and stored for subsequent analysis (Table S2); MALDI‐TOF identification of ethanolic extractions from each isolate indicated that 46% (46 of 100, quality score of 1.85–2.24) were *C*. *coli* , 39% (39 of 100, quality score of 1.91‐2.53) were *C*. *jejuni* , 4% (4 of 100, quality score of 2.01–2.17) were *C*. *lari* with no definitive identification of the remaining 11% (11 of 100, putative quality score < 1.87) (Table S2).

**FIGURE 2:**
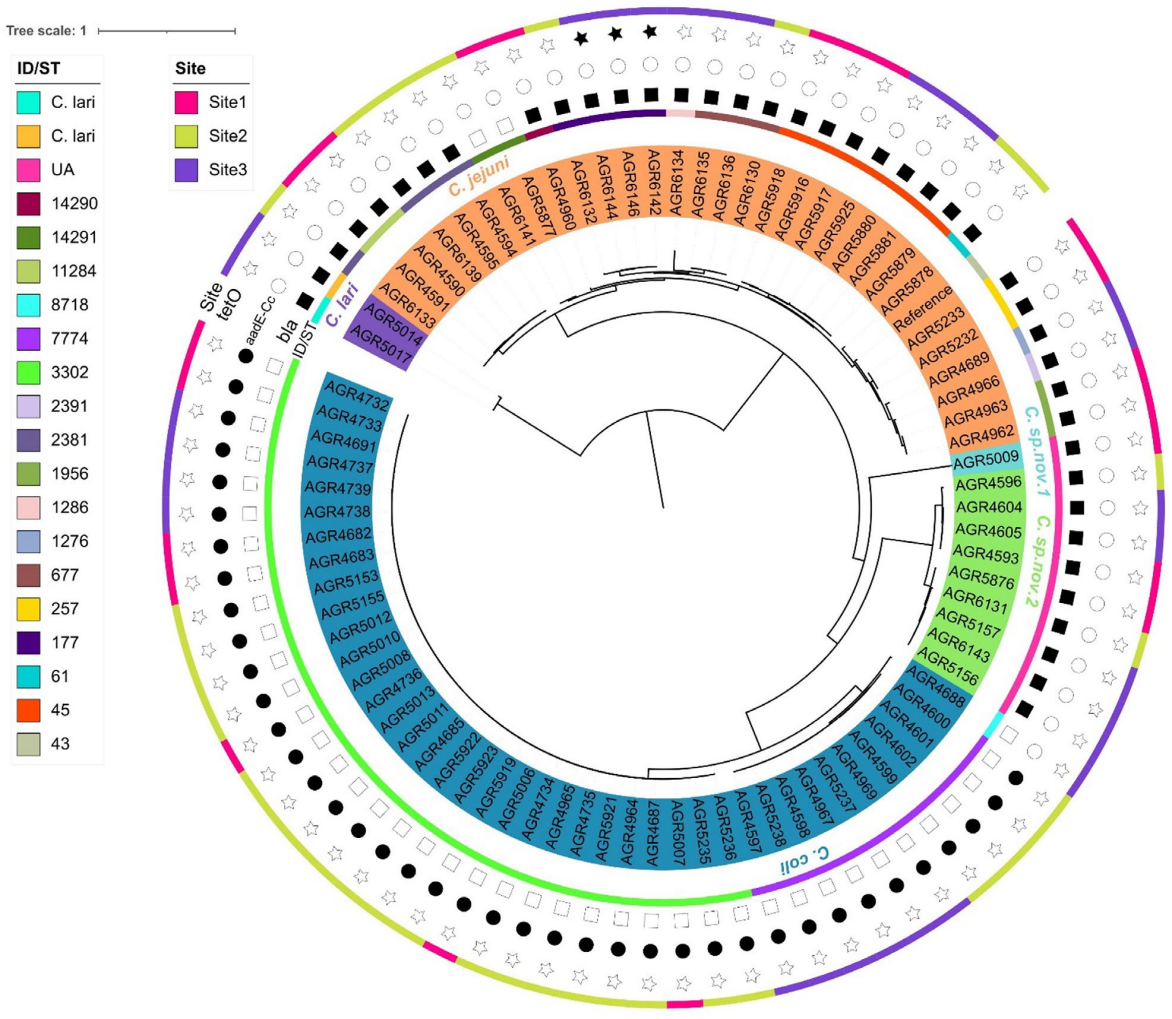
Maximum‐likelihood tree, visualised using the Interactive Tree of Life tool and rooted at the mid‐point, of the core genome single nucleotide polymorphism phylogeny of *Campylobacter* isolates (*n* = 85) isolated from freshwater. The genetic diversity of *Campylobacter* isolates were examined by generating core SNP alignments using Snippy (v. 4.3.6) with *C*. *jejuni* NCTC11168 (accession AL111168) as the reference genome. The Jukes‐Cantor substitution model was used to undertake evolutionary analysis of the core SNP alignment and a phylogenetic tree was inferred by maximum likelihood using IQ‐Tree with the resultant tree imported into the Interactive Tree of Life tool as a Newick file. Clustering broadly separated *Campylobacter* isolates into discrete phylogenies (innermost coloured circle) representing *C*. *jejuni* , *C*. *coli* , *C. lari* and two novel lineages (*Campylobacter* sp. nov. 1, AGR5009) and *Campylobacter* sp. nov. 2 (*n* = 9). Moving outward, the next ring represents multi locus ST, presence/absence of *bla* (beta‐lactamase resistance gene), *aad*E‐Cc (streptomycin resistance gene), and *tet*O (tetracycline resistance gene), with sample site origin as the outermost ring. UA—*Campylobacter* isolates with ST unassigned. The scale bar represents the number of substitutions per site.

A total of 84 isolates were selected for WGS: 41 *C*. *coli* (48.8%), 31 *C*. *jejuni* (36.9%), 2 *C*. *lari* (2.4%), and 10 *Campylobacter* spp. (11.9%) (Table S3). Isolates were chosen for WGS to ensure that at least one isolate of each species from each sample was included where possible. SNP analysis using 18,273 SNPs (1.06% of average genome size, 1.72 Mb) separated the isolates into phylogenies representing *C*. *jejuni* , *C*. *coli* , and *C*. *lari* , with AGR5009 (*Campylobacter* sp. nov. 1) and the remaining nine *Campylobacter* sp. (*Campylobacter* sp. nov. 2) isolates representing separate *Campylobacter* lineages (Figure 2). Taxonomic classification using Centrifuge (Kim et al. 2016) was unable to definitively provide an identification of the 10 *Campylobacter* spp. isolates (#1 match ‘unclassified’) (Table S3).

Using PubMLST (Jolley, Bray, and Maiden 2018), three STs were identified from *C*. *coli* WGS data; ST3302 (73.2%, 30 of 41), ST7774 (24.4%, 10 of 41), and ST8718 (2.4%, 1 of 41) (Figure 2, Table S4). STs from *C*. *jejuni* were more diverse with at least 11 separate STs assigned; ST45 (22.6%, 7 of 31), ST177 (12.9%. 4 of 31), ST2381 (12.9%, 4 of 31), ST677 (9.7%, 3 of 31), ST257 (9.7%, 2 of 31), ST1956 (9.7%, 2 of 31), ST11284 (9.7%, 2 of 31), and singleton ST61, ST1276, ST1286, and ST2391 (3.2%) (Figure 2, Table S4). Two *C*. *jejuni* (AGR5877 and AGR6141) were single locus variants (SLVs) of ST4508 (175*asp*A750; A63G), ST8398 (1*asp*A750; G111A and G114A) and ST14256 (282*gly*A332; C114T, G120A, T201C, C216T, T483C), and assigned the ST ST14291 upon submission to PubMLST. Similarly, AGR4960 was an SLV of ST4508 (216*glt*A5; G12A, T39C, C72T, T207C, C210T, T225C, G348A, G396A); ST7768 (282*gly*A332; C114T, G120A, T201C, C216T, T483C); and ST14259 (175*asp*A751; A63G), and assigned the ST ST14290. After submission to PubMLST and assessment using the specific *C*. *lari* MLST scheme, the two *C*. *lari* could not be assigned an ST because of missing alleles. AGR5014 is ^Cla^ *adk*55, ^Cla^ *atp*A13, ^Cla^ *gln*A5, ^Cla^ *gly*A4, ^Cla^ *pgi*60, ^Cla^ *pgm*138, and ^Cla^ *tkt* unassigned whilst AGR5017 is ^Cla^ *adk*79, ^Cla^ *atp*A59, ^Cla^ *gln*A5, ^Cla^ *gly*A10, ^Cla^ *pgi* unassigned, ^Cla^ *pgm*7, and ^Cla^ *tkt*35. The STs for the 10 *Campylobacter* spp. isolates were unassigned according to the *C*. *jejuni* /coli MLST database; for AGR5009, only the *gln*A allele generated a partial match to *gln*A166 (44 SNPs) from the reference database, an exact match for *asp*A434 for AGR4596 (*Campylobacter* sp. nov. 2), and for the other eight other *Campylobacter* sp. nov. 2 isolates, there was a partial match to only *gly*A343 (four or eight SNPs).

Although no sampling occurred in April and May 2020 (southern hemisphere autumn) during the 13‐month study, due to COVID restrictions, preliminary analysis of seasonal effects on the isolation of the STs suggested that *C*. *jejuni* were more commonly isolated in warmer summer and autumn seasons (71.8%), especially the more generalist STs (ST45, ST61, ST177, ST257, and ST677) where 15 of 17 were isolated in summer and autumn. Conversely 16.1% *C*. *coli* were isolated in summer and autumn (Fisher's exact test, *p* < 0.0001).

**FIGURE 3:**
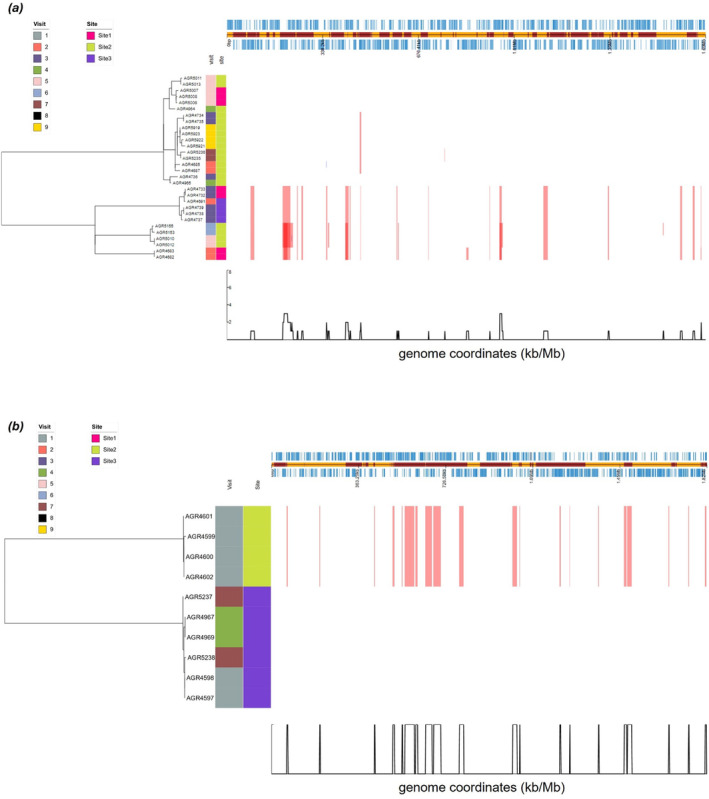
*Campylobacter coli* ST3302 (a) and ST7774 (b) recombination events. Phandango plot of recombination detected with Gubbins with AGR4682 (ST3302) and AGR4597 (ST7774) used as reference sequences, respectively. Site and Visit are annotated in metablocks. Recombination blocks span the taxa in which they are detected, and the region of genes affected in the reference. Red blocks affect *n* > 1, blue blocks affect *n* = 1. Overlapping blocks increase the density of the colour. A sliding window of the number of recombination events affecting any one position in the reference is plotted at underneath.

Preliminary SNP analysis of core genomes from the full isolate set (*n* = 84) revealed that two ST2381 isolates obtained in March 2020 (AGR4595) and March 2021 (AGR6319) from Site 2 differed by only six SNPs. There was also significant similarity between *Campylobacter* sp. nov. 2 isolates; AGR5156/AGR5157 (October 2020) and AGR6143 (March 2021) from Site 3 were identical at the core genome SNP level (0 SNPs), and AGR5877 (Site 1, December 2020) and AGR6141 (Site 2, March 2021) were also identical (0 SNPs). Using core genome SNP analysis from the 84 isolates which underwent WGS, initial analysis indicated that the 30 *C*. *coli* ST3302 were very similar (< 20 SNPs) and were identified from multiple visits to all three sites. Similarly, the 10 *C*. *coli* ST7774 were very similar (< 62 SNPs) identified from multiple visits to Sites 2 and 3. Additional core genome SNP analysis and pairwise SNP comparisons of WGS data from the individual ST3302 (*n* = 21) or ST7774 (*n* = 10) isolates was undertaken using Snippy to provide enhanced phylogenetic resolution using AGR4682 (ST3302) and AGR4597 (ST7774) as reference sequences, respectively. ST3302 isolates were isolated from water samples on seven separate sampling dates, and from all three sites on two individual sampling dates (Figure 3a) and were at least 99.8% similar at the core genome SNP level (total SNPs, 3273, 1.71 Mb genome size). Although there was clear indication of clonality with less than 20 SNPs separating six isolates, other isolates were more dissimilar and differed by up to 2774 SNPs. For ST7774, the 10 isolates were at least 99.91% similar (total SNPs, 1658, 1.82 Mb genome size) and were represented by two distinct clones; six were identified from Site 3 on three separate sampling dates (Figure 3b) with < 6 SNP variation. The other four isolates were separated from the AGR4597 reference by 1649 to 1651 SNPs, representing a different clone. These four identical isolates were recovered from a single water sample from Site 2 (about 600 m from Site 3).

Recombination events separated isolates within ST3302 or ST7774 (Figure 3a,b) and indicated within‐ST variation at the genome level was influenced by recombination events of length from < 100 bp up to almost 40 kb. By mapping recombination co‐ordinates back to the reference genomes, the recombination sites were able to be located and the associated coding sequences identified. For example, one recombination region categorised by Gubbins in ST3302 impacted *gps*A (glycerol‐3‐phosphate dehydrogenase), *gat*B (aspartyl/glutamyl‐tRNA (Asn/Gln) amidotransferase subunit B), and *lux*S (S‐ribosylhomocysteine lyase, a quorum sensing auto‐inducer) (Ramić et al. 2023), and another the *pan*BCD (vitamin B_5_ biosynthesis) (Sheppard et al. 2013) and *cdt*ABC (cytolethal distending toxin) operons. Within ST7774, the LPS biosynthesis region including the *pgl* (protein glycosylation) operon, and another including *fli*G (flagellar motor switch protein) were identified as within recombination regions.

**FIGURE 4:**
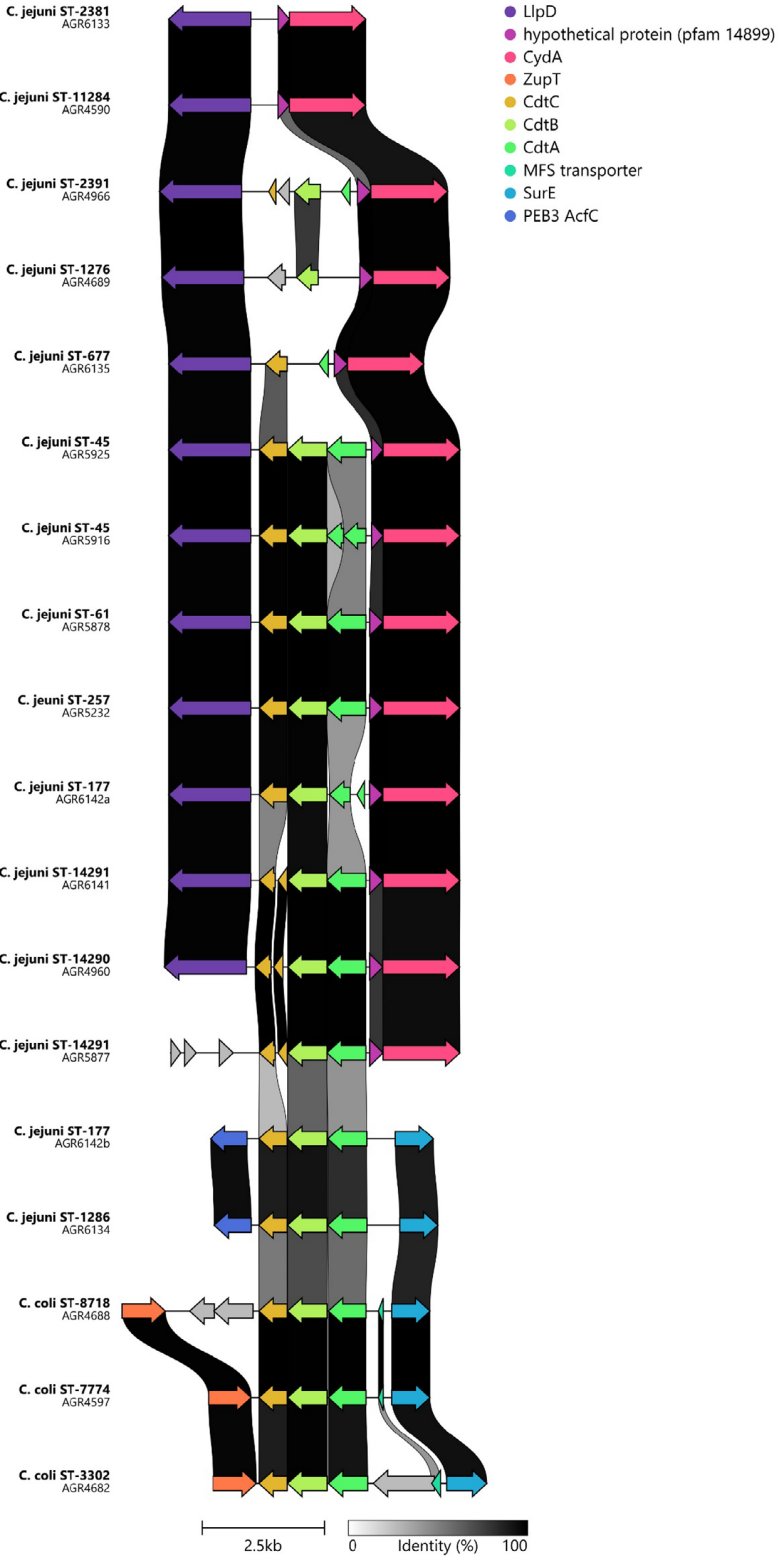
Comparative analysis of the *cdt*ABC region, encoding the cytolethal distending toxin, from *C*. *jejuni* and *C*. *coli* whole genome sequence data demonstrating recombination impacts. *C*. *coli* associated *cdt*ABC flanking regions differed from those associated with *C*. *jejuni* with evidence of complete replacement or total loss of the *cdt*ABC operon. AGR6142a and AGR6142b represent the two separate *cdt*ABC regions in AGR6142. UA— *C*. *jejuni* isolates with ST unassigned. Figure was prepared using Clinker.

Using WGS data from *C*. *jejuni* and *C*. *coli* , the *cdt*ABC operon encoding for the cytolethal distending toxin was examined in more detail with alignments of approximately 6 kb including *cdt*ABC flanking regions to ascertain the potential impact of recombination events with evidence of complete replacement or total loss of *cdt*ABC operon across different *Campylobacter* lineages (Figure 4). The *cdt*ABC operon was complete in all *C*. *coli* and most *C*. *jejuni* (17 of 31, 54.8%), present in all ST45 (*n* = 7) isolates, ST177 (*n* = 4), ST257 (*n* = 2), SLV ST4508 (*n* = 2), ST61 (*n* = 1), and ST1286 (*n* = 1). Analysis of the remaining isolates indicated the presence of a disrupted operon in *C*. *jejuni* ST2381 (*n* = 4), ST1956 (*n* = 2), ST11284 (*n* = 2), ST1276 (*n* = 1), ST2391 (*n* = 1), and the SLV ST4508/ST7768 isolate (*n* = 1), or in ST677 (*n* = 3) where the complete *cdt*ABC operon was absent. Notably the four ST177 isolates had two copies of the *cdt*ABC operon; the *cdt*A gene in the *C*. *jejuni* ‐associated region was impacted by a point mutation leading to a premature stop codon, but the *C*. *coli* ‐associated *cdt*ABC region was intact. Furthermore, although the *C*. *jejuni* ‐associated operon was absent in the single ST1286 isolate, an intact *C*. *coli* operon was evident.

Antimicrobial sensitivity testing was undertaken to examine the susceptibility of the *Campylobacter* isolates to several distinct antibiotic classes including nalidixic acid and ciprofloxacin (fluoroquinolones), penicillin G and ampicillin (penicillins), gentamicin (aminoglycoside), erythromycin (macrolide), and tetracycline (Table S5). All isolates examined (*n* = 99) were sensitive to nalidixic acid, ciprofloxacin, gentamycin, and erythromycin. Almost all isolates were susceptible to ampicillin (96.0%) and almost all isolates were sensitive to tetracycline (96.0%) but almost all (98.0%) were resistant to penicillin G (Table S5); the remaining isolates ( *C*. *jejuni* AGR4691 and *C*. *lari* AGR5015) displayed intermediate resistance.

**FIGURE 5:**
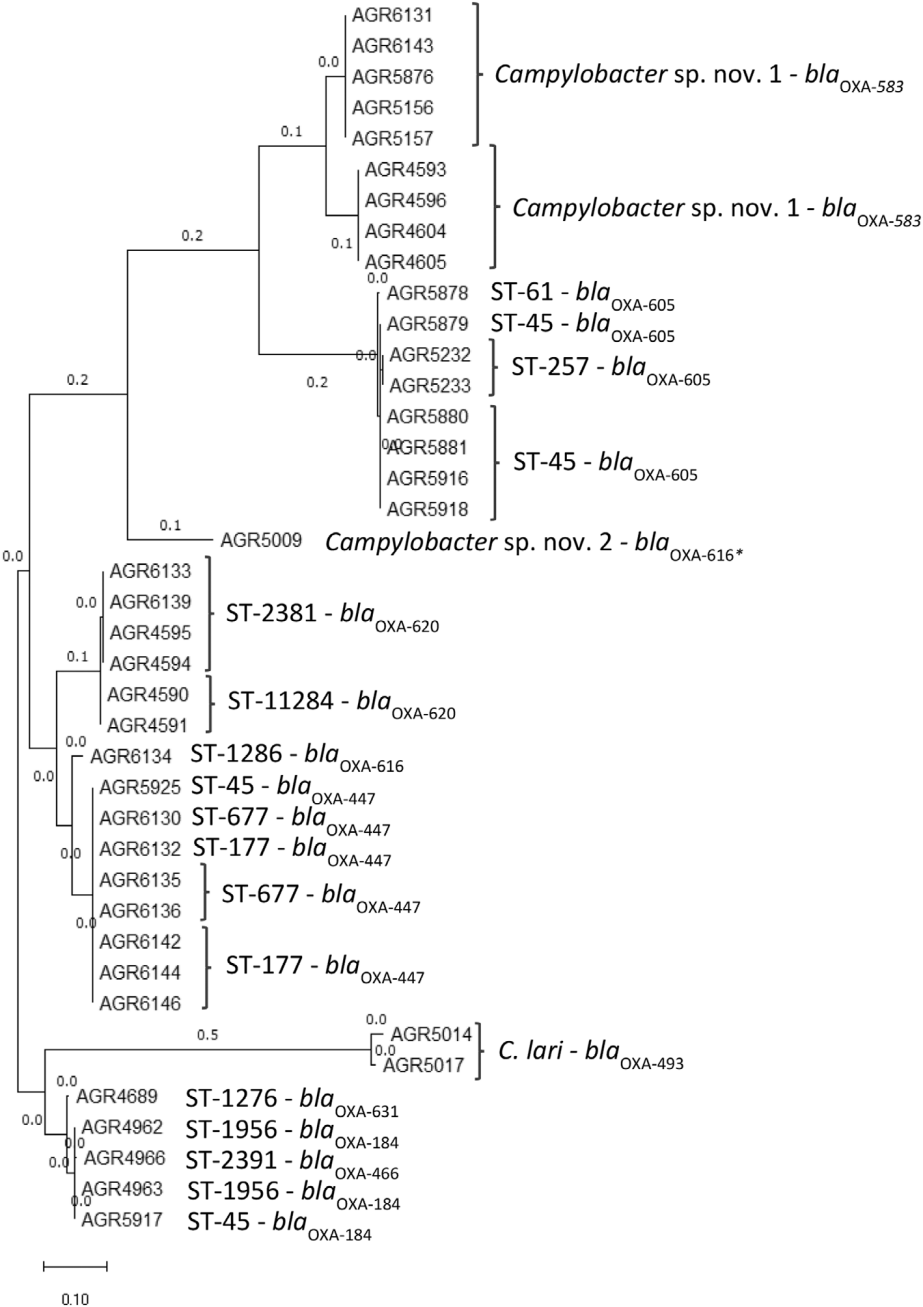
Evolutionary analysis of *bla*_OXA_ genes identified from *C*. *jejuni* and *Campylobacter* sp. (AGR5009) isolated from freshwater, by maximum likelihood method. The evolutionary history was inferred by using the maximum likelihood method and JTT matrix‐based model. The tree with the highest log likelihood (−2679.78) is shown. Initial tree(s) for the heuristic search were obtained automatically by applying Neighbour‐Join and BioNJ algorithms to a matrix of pairwise distances estimated using the JTT model, and then selecting the topology with superior log likelihood value. The tree is drawn to scale, with branch lengths measured in the number of substitutions per site (next to the branches). This analysis involved 40 amino acid sequences. There are a total of 260 positions in the final alignment. Evolutionary analyses were conducted in MEGA11. * *bla*_OXA_ from AGR5009 was < 80% similar to *bla*_OXA‐616_ at the amino acid sequence level.

The *aad*E‐Cc gene that encodes an aminoglycoside 6‐adenylyltransferase for streptomycin resistance was the only antimicrobial resistance‐associated genetic marker identified from *C*. *coli* , found in 97.6% (40 of 41) isolates (Table S4). There was concordance between the presence of the *tet*O gene encoding tetracycline resistance ribosomal protection protein (TetO) from *C*. *jejuni* ST177 (*n* = 3), and the tetracycline resistant phenotype present in the same isolates recovered from the same water sample (Table S4). Ampicillin resistance was associated with isolates having the *bla*_OXA‐493_ ( *C*. *lari* AGR5014 and AGR5017) and *bla*_OXA‐184_ (ST1956, AGR4962, and AGR4963) genes (Table S4). The only remaining antimicrobial resistance‐associated genetic markers identified were 10 class D beta‐lactamase (bla_OXA_) protein sequences from *C*. *jejuni* and *Campylobacter* spp. which generally clustered according to ST (Figure 5), and which were absent in *C*. *coli* .

## 4 Discussion

Several New Zealand studies have closely examined the source attribution of *Campylobacter* as a causative agent of human gastrointestinal illness with specific risk factors including the preparation and consumption of poultry products (Lake et al. 2021; Liao et al. 2019; Harrison et al. 2021). However, point‐source environmental contamination events have also caused large waterborne outbreaks of disease, most notably the Havelock North outbreak of campylobacteriosis caused by contamination of bore drinking water with sheep faecal material after a heavy rainfall event (Gilpin et al. 2020). Furthermore, thermophilic *Campylobacter* represent a public health concern as they are commonly isolated from freshwater sites impacted by faecal contamination from humans, animals or wildlife and have been implicated as a risk factor (recreational water contact) for human campylobacteriosis (Shrestha et al. 2019; Carter et al. 2009; Nohra et al. 2016; Irshad et al. 2016).

Water managers use *E*. *coli* as an indicator of faecal contamination to assess human health risk associated with freshwater recreational contact as other waterborne zoonotic pathogens, such as *Campylobacter*, are more fastidious in their growth requirements (Till et al. 2008; Devane et al. 2020). However, with the advent of high‐resolution WGS methods, some naturalised *E*. *coli* from non‐recent faecal contamination events able to be stably maintained in the environment, have been identified (Cookson et al. 2022; Byappanahalli et al. 2006; Berthe et al. 2013; Touchon et al. 2020). Other isolates phenotypically indistinguishable from *E*. *coli* by conventional water quality assessment methods, have also been isolated and characterised as distinct *Escherichia* species commonly associated with poultry, wildlife and the environment (Liu et al. 2015; van der Putten et al. 2021; Gilroy et al. 2021). Previous studies have noted thermophilic *Campylobacter* STs isolated from freshwater and wildlife, absent from human campylobacteriosis disease cases (Shrestha et al. 2019; Carter et al. 2009; Nohra et al. 2016; French et al. 2009), but investigations to ascertain their impact on human health risk assessments have not been determined. As with naturalised *E*. *coli* , it is only through molecular analysis that the human health risk of individual *Campylobacter* isolates can easily be differentiated to provide more accurate freshwater assessments of whether isolates are naturalised. Host niches for different *Campylobacter* STs have been widely reported with some lineages represented by ST45, ST61, ST257, and ST677, detected in this study (Table S6), widely dispersed geographically, with multiple sources and associated with human disease (Dearlove et al. 2016; Méric et al. 2018; Cody et al. 2012; Kwan et al. 2008; Wysok et al. 2020; Harvala et al. 2016; Kovanen et al. 2016). Other STs such as ST1276, ST1286 and ST1956 are much less commonly reported, and although isolated from multiple geographical regions, are rarely isolated from humans, but more commonly isolated from wild birds and poultry (Table S6). Some *C*. *jejuni* (ST2381, ST2391, ST11284) and *C*. *coli* (ST3302, ST7774, ST8178) from this study, appear to be geospatially isolated, detected only in New Zealand (Table S6) specifically from freshwater or wild birds (Shrestha et al. 2019; Carter et al. 2009; Nohra et al. 2016). Isolates representative of these environmental STs can be taxonomically designated to *C*. *jejuni* and *C*. *coli* , but the genus *Campylobacter* has become increasingly complex with host specific and environmental taxa characterised as WGS and comparative genomics methods have been applied to isolates obtained from less well‐established animal or environmental hosts (Mourkas et al. 2021; Miller et al. 2024). Ten isolates from this study are genetically distinct from currently described thermophilic *Campylobacter*, were unable to be readily defined taxonomically using phenotypic/structural (MALDI‐TOF) or genomic methods and upon phylogenetic analysis were separated into two likely new species represented by one and nine isolates, respectively. These freshwater isolates were also unable to be typed using conventional multi‐locus sequencing of generic *Campylobacter jejuni* /*coli* loci. Thus, like *E*. *coli* , it appears that there may be two types of naturalised *Campylobacter* isolated from freshwater in New Zealand; isolates readily identified as *C*. *jejuni* (ST2381, ST2391, ST11284) and *C*. *coli* (ST3302, ST7774) which have not to date been associated with human campylobacteriosis, and naturalised *Campylobacter* isolates representative of new species which are environmentally associated. The geographical isolation of New Zealand and unique biodiversity are likely to drive the evolution of new thermophilic *Campylobacter* lineages and their maintenance in the environment (Wilkinson et al. 2019; Bloomfield et al. 2020). However, in areas impacted by significant anthropogenic activity such as agriculture and human wastewater discharge points, their presence may be replaced by STs more associated with ruminants and human campylobacteriosis (Meinersmann et al. 2019), but not poultry (Meinersmann et al. 2023).

Overall, New Zealand is a low user of antibiotics in livestock industries (Hillerton et al. 2017) with 57% of antibiotic sales for use in dairy cattle (Anon 2023). The low prevalence of antimicrobial resistant bacteria and antimicrobial resistance genes recovered from the carcasses of pigs, poultry, very young calves and dairy cattle suggests that AMR in food animals poses a limited public health risk and reflects low use in New Zealand animal production systems (Cornelius et al. 2024). Only 16.6% of *Campylobacter* were resistant to quinolones and tetracycline with the remaining isolates susceptible to all tested antimicrobials (gentamycin, streptomycin, ciprofloxacin, erythromycin, nalidixic acid, and tetracycline) (Cornelius et al. 2024). However, the emergence in New Zealand of a lineage of *Campylobacter* ST6964 resistant to tetracycline and fluoroquinolones has been observed in poultry and ST6964 was also associated with human infection (French et al. 2019).

Apart from intrinsic penicillin resistance associated with the presence of beta‐lactamases, overall, the prevalence of AMR genes in isolates from this study was relatively low, with only *tetO* and *aad*E‐Cc detected. Previous studies have been undertaken to isolate and characterise *Campylobacter* from freshwater with the aim of addressing human health risk from recreational contact, but few have taken a WGS approach to examine antimicrobial resistance phenotype and genotype combinations. In Brazil, several *C*. *coli* isolated from freshwater were tetracycline resistant and were *tetO*‐positive upon WGS (Gomes et al. 2023). Other studies have used a PCR‐based approach to identify antimicrobial resistance genes such as *tetO* (Andrzejewska et al. 2022; Chibwe, Odume, and Nnadozie 2024). The *tetO* gene is commonly associated with the tetracycline resistance phenotype in *Campylobacter* (French et al. 2019; Zarske et al. 2024; Rivera‐Mendoza et al. 2020; Schiaffino et al. 2024) and in this study the presence of *tetO* in the *C*. *jejuni* ST177 isolates was associated with a tetracycline resistance phenotype. The *aad*E‐Cc gene associated with a aminoglycoside/streptomycin resistant phenotype in *C*. *coli* has been identified previously in human, monkey and sewage samples from Brazil (Gomes et al. 2023) and human diarrhoeal samples from Poland (Wysok et al. 2020), and was present in all *C*. *coli* ST3302 and ST7774 isolates, but not the single ST8178 isolate. The ampicillin resistant phenotype of freshwater ST1956 (AGR4962 and AGR4963) and a single ST45 isolate (AGR5917) was associated with the presence of *bla*_OXA‐184_ and has previously been identified in *C*. *jejuni* isolated from human diarrhoeal disease in Japan (Morita et al. 2023). However, no definitive genotype could be identified which was associated with the ampicillin resistant phenotype in the two *C*. *lari* isolates. Concordance between phenotype and genotype can be influenced by computational pipelines, level of genome coverage and antimicrobial resistance gene type (Hodges et al. 2021) necessitating the requirement for describing pipelines, tool and database versions for all reporting of data.

With this study, there was a clear relationship between the freshwater *E*. *coli* concentrations, Sites, total numbers of ruminants upstream, and urban/built areas. Land‐use can have a significant impact on the types of faecal sources and the associated pathogens contaminating freshwater catchments (Phiri et al. 2020b) where catchments with saturated soils, a high percentage of pasture, and high rainfall have the greatest prevalence of *Campylobacter* and the highest abundance of *E*. *coli* in waterways (Phiri et al. 2020b). Further work undertaken in New Zealand suggested a positive association and high prevalence of thermophilic *Campylobacter* isolated from freshwater samples impacted by dairy land‐use (Cookson et al. 2024) and that presence of *Campylobacter* was positively associated with microbial source tracking markers specific for avian and ruminant faecal sources (Cookson et al. 2024). Source attribution of *Campylobacter* isolated from surface water samples in Europe was linked to wild birds ( *C*. *jejuni* , 60.0%; *C*. *coli* , 93.7%) and broilers ( *C*. *jejuni* : 18.9%; *C*. *coli* : 5.6%) (Mulder et al. 2020). The intermittent nature of faecal sources was characterised by the detection of ST45 isolates, previously associated with human, poultry, cattle and sheep (Lake et al. 2021), from all three sites on only one or two consecutive sampling occasions. Other STs more associated with human disease, such as ST61, ST177, ST257 and ST657 were uncommon and only isolated from freshwater samples on one or two visits. Although the catchment for Site 1 was represented in part by urban land‐use, no specific links with isolates was possible, apart from the detection of two ST257, a generalist ST found in Europe, Asia and Oceania, commonly associated with human infection (Wysok et al. 2020) and cattle (Kwan et al. 2008), on one visit. In contrast, ST2381 isolates previously isolated from wild birds (including pūkeko, *Porphyrio melanotus*, native water rails), and freshwater samples (Phiri et al. 2020a; Shrestha et al. 2019; Nohra et al. 2016; Irshad et al. 2016), including the Manawatū River catchment (Carter et al. 2009), were only recovered from samples collected at Site 2 on three visits, but over the full 13‐month duration of the study. Furthermore, core genome analysis (*n* = 84 genomes) indicated only a six SNP difference between two ST2381 isolates from the first (March 2020) and last sampling (March 2021) potentially due to waterborne contamination from pūkeko which were observed during several sites visits. Several *Campylobacter* sp. nov. 2 isolates identified from water samples collected at different sites on different visits also appeared clonal suggesting a common source. Similarly, the frequency at which *C*. *coli* ST3302 and ST7774, previously isolated from wild birds and surface/untreated drinking water supplies (Phiri et al. 2020a; Nohra et al. 2016) were isolated over the study suggest a local wildlife source and continual faecal shedding in all three catchments throughout the study. Although the host for *Campylobacter* sp. nov. 2 has not been identified, the nine representative isolates of this lineage were isolated from all three catchments over the 13‐month duration of the study indicating a continuous local source of contamination.

*Campylobacter* species are well‐recognised for both host specific and multi‐host generalist lineages with much of the genome plasticity due to horizontal gene transfer which brings about adaptation more rapidly than individual SNPs (Biggs et al. 2011; Sheppard et al. 2013; Mourkas et al. 2021). The transfer of genetic material can bring about new adaptive trajectories which allow for zoonotic transfer or AMR, but whether the new genotype thrives is dependent on availability of DNA for recipient cells, cell‐dependent factors which may obstruct homologous recombination, and finally the impact of the recombination event on recipient cell in the receiving environment (Mourkas et al. 2021). Previous comparative studies on the contrasting roles of individual point mutations versus recombination events in human and poultry *C*. *jejuni* ST474 suggested that while the rates of recombination and mutation are similar, recombination has a greater impact with respect to genome coverage (Biggs et al. 2011). The source of recombined DNA is more likely to come from *C*. *jejuni* strains closely related to ST474 and implies an enhanced role for recombination in the emergence of new lineages and generating divergence (Biggs et al. 2011).

Compared to the complete core genome SNP analysis across all 84 isolates from within this study, there was an increase in number of core genome SNPs identified from individual pairwise comparisons with the individual *C*. *coli* ST (ST3302, *n* = 30; or ST7774, *n* = 10) analyses. Subsequent investigation of the core genome SNP sequence with Gubbins indicated *C*. *coli* genome dissimilarities due to recombination events. Recombination events impacted at least 43 and 26 loci regions in ST3302 and ST7774, respectively with average tract lengths of ~5 kb and ~7 kb. Many recombination regions were intergenic, or in genes with unknown function, however other recombination tracts contained genes involved in quorum sensing (*lux*S), pantothenate (vitamin B_5_) biosynthesis pathway (*pan*BCD), and cytolethal distending toxin (*cdt*ABC) in ST3302, and the N‐glycosylation pathway (*pgl* operon), the flagella switch protein (*flg*E) and the *lut*AB region in ST7774. Of note was that the *pan*BCD and *cdt*ABC regions were associated with the same 14.8 kb recombination region in *C*. *coli* ST3302. Previous studies have described host specificity within the *pan*BCD genomic region where improved growth of *pan*BCD‐positive cattle isolates in vitamin B_5_‐depleted media occurred indicating that this difference may be an adaptation to host diet (Sheppard et al. 2013). The cytolethal distending toxin is thought to be involved in disruption of host cell division (Whitehouse et al. 1998) and studies to investigate the *cdt*ABC prevalence using molecular methods have demonstrated that PCR is inadequate to confirm the presence of the *cdt*ABC operon due to sequence divergence variants (Guirado et al. 2022). However, other recent work taking a WGS approach to analyse virulence factors of 129 *Campylobacter* from clinical samples identified the *cdt*ABC operon in 82.9% of sequenced isolates (Quino et al. 2022).

Detailed examination of the *cdt*ABC region from *C*. *jejuni* and *C*. *coli* in this study provides clear evidence of significant recombination activity and complete or partial loss of the operon in some environmental‐associated STs (ST677, ST1276, ST2381, ST2391, ST11284), a defective *cdt*A gene (ST45, *n* = 2; ST177) or defective *cdt*C gene (ST4508, SLV ST4508, SLV ST4508/ST7768). Only *C*. *jejuni* lineages with an established risk profile for human disease, such as ST45 (*n* = 5), ST61 and ST257 possessed intact *cdt*ABC genes. The *cdt*ABC operon associated with *C*. *coli* is flanked by different genes suggesting a different genome location compared to the *C*. *jejuni* *cdt*ABC homologue. Intriguingly ST177 isolates with a defective *cdt*A gene (Figure 4; AGR6142a) also had an intact *C*. *coli* ‐associated *cdt*ABC operon (Figure 4, AGR6142b) inserted at a separate chromosomal site, and *C*. *jejuni* ST1286 only possessed the *C*. *coli* operon. These data provide further evidence of the decay of loci involved in human disease in *C*. *jejuni* STs with wild bird and/or environmental ecologies and interspecies gene flow (introgression) between *C*. *coli* and *C*. *jejuni* , including in some *C*. *jejuni* isolates where the *C*. *jejuni* ‐associated *cdt*ABC operon is non‐functional. However, the beneficial traits associated with the intact *cdt*ABC operon and enhanced environmental fitness in *C*. *coli* is uncertain.

## 5 Conclusion

Waterbodies are frequently contaminated by run‐off from various faecal sources, leading to the mixing and transport of both pathogenic and non‐pathogenic contaminants from diverse origins (Cookson et al. 2022, 2024). *E*. *coli* are routinely used as indicators of faecal contamination and serve as surrogates for other waterborne pathogens (Devane et al. 2020). However, the use of WGS techniques has proven to be a crucial tool in providing enhanced resolution to differentiate *E*. *coli* from recent faecal contamination events (Cookson et al. 2024), naturalised *E*. *coli* that have adapted for enhanced environmental survival and persistence (Byappanahalli et al. 2006; Berthe et al. 2013; Touchon et al. 2020), and non‐ *E*. *coli* Escherichia species that are unlikely to cause frequent human illness (Liu et al. 2015; van der Putten et al. 2021; Gilroy et al. 2021). This study illustrates a similar ‘naturalised’ concept with environmental *Campylobacter*, including previously undescribed *Campylobacter* species, as well as *C*. *jejuni* and *C*. *coli* STs absent from clinical disease. These STs appear to be attenuated in some loci associated with human pathogenesis due to recombination. Longitudinal studies, high‐resolution molecular analysis, and enumeration of waterborne *Campylobacter* from sites with contrasting land use will be essential to provide water managers with the necessary information to conduct accurate human health risk assessments. Without such studies, distinguishing environmental sources of naturalised *Campylobacter* may remain challenging, thus impeding their relevance to water quality and health risk assessments and hindering informed decision‐making on land‐use mitigation strategies for improved water quality management.

## Author Contributions

**Adrian L. Cookson:** conceptualization, methodology, validation, formal analysis, investigation, data curation, writing – original draft, visualization, supervision, project administration, funding acquisition. **Sara Burgess:** methodology, software, formal analysis, writing – review and editing. **Anne C. Midwinter:** methodology, writing – review and editing. **Jonathan C. Marshall:** conceptualization, methodology, software, validation, formal analysis, resources, data curation, writing – review and editing, visualization. **Marie Moinet:** conceptualization, methodology, validation, investigation, data curation, supervision, project administration, writing – review and editing. **Lynn Rogers:** investigation, writing – review and editing. **Ahmed Fayaz:** validation, investigation, data curation, writing – review and editing. **Patrick J. Biggs:** conceptualization, methodology, software, validation, formal analysis, data curation, resources, writing – review and editing, visualization. **Gale Brightwell:** writing – review and editing, project administration, funding acquisition.

## Conflicts of Interest

The authors declare no conflicts of interest.

## Supporting information


**TABLE S1.** Catchment delineation and land‐use identification for freshwater sampling sites. Land cover data (%) for each of the three sites was sourced from the Land Cover Database version 5.0, Mainland, New Zealand*.
**TABLE S2**. Bacterial strains (*n* = 100) isolated in this study and preliminary identification using MALDI‐TOF.
**TABLE S3**. Whole genome sequencing assembly details and identification of *Campylobacter* (*n* = 84).
**TABLE S4**. MLST sequence types and antimicrobial resistance genes identified from Campylobacter (n = 84) whole genome sequence data. *UA—unassigned using *C. coli* / *C. jejuni* MLST scheme, # bla_OXA_ from AGR5009 was < 80% similar to bla_OXA‐616_ at the amino acid sequence level.
**TABLE S5**. Antibiotic sensitivity testing zone sizes (mm).
**TABLE S6**. Source attribution and geographical distribution data associated with *Campylobacter* sequence types (ST) identified in this study. Data obtained from PubMLST (July 2024). ‘Human’ totals include human stool, human blood culture and human unspecified; ‘Cattle’ totals include cattle, beef offal or meat, cattle faeces and calf; ‘Sheep’ totals include sheep, lamb, lamb offal or meat and sheep faeces; ‘Chicken’ totals include chicken and chicken offal or meat.

## Data Availability

Whole genome sequencing short‐read data are openly available under NCBI BioProject number PRJNA1137954 (SAMN42652974‐SAMN42653057).
