# Supplementary material for: New Campylobacter Lineages in New Zealand Freshwater: Pathogenesis and Public Health Implications
Source: Environ Microbiol. 2024 Dec 16;26(12):e70016. doi: 10.1111/1462-2920.70016 (PMC11649337; doi:10.1111/1462-2920.70016)
Supplement: Supplementary file 1 — TABLE S1. Catchment delineation and land‐use identification for freshwater sampling sites. Land cover data (%) for each of the three sites was sourced from the Land Cover Database version 5.0, Mainland, New Zealand*. TABLE S2. Bacterial strains (n = 100) isolated in this study and preliminary identification using MALDI‐TOF. TABLE S3. Whole genome sequencing assembly details and identification of Campylobacter (n = 84). TABLE S4. MLST sequence types and antimicrobial resistance genes identified from Campylobacter (n = 84) whole genome sequence data. *UA—unassigned using C. coli / C. jejuni MLST scheme, # blaOXA from AGR5009 was < 80% similar to blaOXA‐616 at the amino acid sequence level. TABLE S5. Antibiotic sensitivity testing zone sizes (mm). TABLE S6. Source attribution and geographical distribution data associated with Campylobacter sequence types (ST) identified in this study. Data obtained from PubMLST (July 2024). ‘Human’ totals include human stool, human blood culture and human unspecified; ‘Cattle’ totals include cattle, beef offal or meat, cattle faeces and calf; ‘Sheep’ totals include sheep, lamb, lamb offal or meat and sheep faeces; ‘Chicken’ totals include chicken and chicken offal or meat. [file EMI-26-e70016-s001.docx]

***Appendix***

***Table S1.*** Catchment delineation and land-use identification for freshwater sampling sites. Land cover data (%) for each of the three sites was sourced from the Land Cover Database version 5.0, Mainland, New Zealand*.

| Site | Total area, ha | bare, ha (%) | built, ha  (%) | crops, ha (%) | grass, ha  (%) | shrub & scrub, ha (%) | trees, ha (%) | water, ha (%) | Catchment length (km) | Dairy (n) u/s^#^ | Beef (n) u/s | Sheep (n) u/s |
| --- | --- | --- | --- | --- | --- | --- | --- | --- | --- | --- | --- | --- |
| Site 1 Tapuata | 1592.3 | 9.1 (0.57) | 164.2 (10.3) | 0 (0) | 1373.4 (86.3) | 1.2 (0.1) | 36.1 (2.3) | 8.3 (0.5) | 11.7 | 1310 | 525 | 4687 |
| Site 2 Mangatera | 10073.1 | 22.5 (0.2) | 335.6 (3.3) | 6.8 (0.1) | 9287 (92.2) | 6.6 (0.1) | 402.2 (4.0) | 12.3 (0.1) | 24.9 | 8807 | 3426 | 29000 |
| Site 3 Makirikiri | 970.4 | 0.1 (0.01) | 9.4 (1.0) | 0 (0) | 933.7 (96.2) | 0 | 24.3 (2.5) | 3 (0.3) | 8.4 | 1028 | 340 | 3041 |

* LCDB v5.0 - Land Cover Database version 5.0, Mainland, New Zealand (2020) <https://lris.scinfo.org.nz/layer/104400-lcdb-v50-land-cover-database-version-50-mainland-new-zealand/>

# Number of dairy cattle associated with catchment found upstream (u/s) of the respective sample site.

***Table S2.*** Bacterial strains (n=100) isolated in this study and preliminary identification using MALDI-TOF. * NOIP - no organism identification possible

| ***Isolate*** | ***Site*** | ***Visit*** | ***Date*** | ***MALDI-TOF Organism*** | ***MALDI-TOF Score*** |
| --- | --- | --- | --- | --- | --- |
| AGR4590 | Site 1 | 1 | 2/03/2020 | *Campylobacter jejuni* | 2.01 |
| AGR4591 | Site 1 | 1 | 2/03/2020 | *Campylobacter jejuni* | 1.91 |
| AGR4593 | Site 1 | 1 | 2/03/2020 | NOIP* | 1.34 |
| AGR4594 | Site 2 | 1 | 2/03/2020 | *Campylobacter jejuni* | 2.04 |
| AGR4595 | Site 2 | 1 | 2/03/2020 | *Campylobacter jejuni* | 1.91 |
| AGR4596 | Site 2 | 1 | 2/03/2020 | NOIP | 1.87 |
| AGR4597 | Site 3 | 1 | 2/03/2020 | *Campylobacter coli* | 1.97 |
| AGR4598 | Site 3 | 1 | 2/03/2020 | *Campylobacter coli* | 1.85 |
| AGR4599 | Site 2 | 1 | 2/03/2020 | *Campylobacter coli* | 1.91 |
| AGR4600 | Site 2 | 1 | 2/03/2020 | *Campylobacter coli* | 2.02 |
| AGR4601 | Site 2 | 1 | 2/03/2020 | *Campylobacter coli* | 2.02 |
| AGR4602 | Site 2 | 1 | 2/03/2020 | *Campylobacter coli* | 1.99 |
| AGR4603 | Site 3 | 1 | 2/03/2020 | NOIP | 1.61 |
| AGR4604 | Site 3 | 1 | 2/03/2020 | NOIP | 1.39 |
| AGR4605 | Site 3 | 1 | 2/03/2020 | NOIP | 1.39 |
| AGR4682 | Site 1 | 2 | 8/06/2020 | *Campylobacter coli* | 2.12 |
| AGR4683 | Site 1 | 2 | 8/06/2020 | *Campylobacter coli* | 2.05 |
| AGR4684 | Site 2 | 2 | 8/06/2020 | *Campylobacter jejuni* | 2.43 |
| AGR4685 | Site 2 | 2 | 8/06/2020 | *Campylobacter coli* | 2.17 |
| AGR4686 | Site 2 | 2 | 8/06/2020 | *Campylobacter jejuni* | 2.46 |
| AGR4687 | Site 2 | 2 | 8/06/2020 | *Campylobacter coli* | 2.16 |
| AGR4688 | Site 3 | 2 | 8/06/2020 | *Campylobacter coli* | 2.05 |
| AGR4689 | Site 3 | 2 | 8/06/2020 | *Campylobacter jejuni* | 2.33 |
| AGR4690 | Site 3 | 2 | 8/06/2020 | *Campylobacter jejuni* | 2.31 |
| AGR4691 | Site 3 | 2 | 8/06/2020 | *Campylobacter coli* | 2.16 |
| AGR4732 | Site 1 | 3 | 1/07/2020 | *Campylobacter coli* | 2.16 |
| AGR4733 | Site 1 | 3 | 1/07/2020 | *Campylobacter coli* | 2.20 |
| AGR4734 | Site 2 | 3 | 1/07/2020 | *Campylobacter coli* | 2.20 |
| AGR4735 | Site 2 | 3 | 1/07/2020 | *Campylobacter coli* | 2.06 |
| AGR4736 | Site 2 | 3 | 1/07/2020 | *Campylobacter coli* | 2.12 |
| AGR4737 | Site 3 | 3 | 1/07/2020 | *Campylobacter coli* | 2.24 |
| AGR4738 | Site 3 | 3 | 1/07/2020 | *Campylobacter coli* | 2.17 |
| AGR4739 | Site 3 | 3 | 1/07/2020 | *Campylobacter coli* | 2.08 |
| AGR4740 | Site 3 | 3 | 1/07/2020 | *Campylobacter coli* | 2.16 |
| AGR4960 | Site 1 | 4 | 10/08/2020 | *Campylobacter jejuni* | 2.30 |
| AGR4961 | Site 1 | 4 | 10/08/2020 | *Campylobacter jejuni* | 2.32 |
| AGR4962 | Site 1 | 4 | 10/08/2020 | *Campylobacter jejuni* | 2.37 |
| AGR4963 | Site 1 | 4 | 10/08/2020 | *Campylobacter jejuni* | 2.33 |
| AGR4964 | Site 2 | 4 | 10/08/2020 | *Campylobacter coli* | 2.19 |
| AGR4965 | Site 2 | 4 | 10/08/2020 | *Campylobacter coli* | 2.20 |
| AGR4966 | Site 3 | 4 | 10/08/2020 | *Campylobacter jejuni* | 2.21 |
| AGR4967 | Site 3 | 4 | 10/08/2020 | *Campylobacter coli* | 2.03 |
| AGR4968 | Site 3 | 4 | 10/08/2020 | *Campylobacter coli* | 2.04 |
| AGR4969 | Site 3 | 4 | 10/08/2020 | *Campylobacter coli* | 2.06 |
| AGR5006 | Site 1 | 5 | 1/09/2020 | *Campylobacter coli* | 2.23 |
| AGR5007 | Site 1 | 5 | 1/09/2020 | *Campylobacter coli* | 2.23 |
| AGR5008 | Site 1 | 5 | 1/09/2020 | *Campylobacter coli* | 2.12 |
| AGR5009 | Site 1 | 5 | 1/09/2020 | NOIP | 1.69 |
| AGR5010 | Site 2 | 5 | 1/09/2020 | *Campylobacter coli* | 2.10 |
| AGR5011 | Site 2 | 5 | 1/09/2020 | *Campylobacter coli* | 2.12 |
| AGR5012 | Site 2 | 5 | 1/09/2020 | *Campylobacter coli* | 2.05 |
| AGR5013 | Site 2 | 5 | 1/09/2020 | *Campylobacter coli* | 2.03 |
| AGR5014 | Site 3 | 5 | 1/09/2020 | *Campylobacter lari* | 2.06 |
| AGR5015 | Site 3 | 5 | 1/09/2020 | *Campylobacter lari* | 2.01 |
| AGR5016 | Site 3 | 5 | 1/09/2020 | *Campylobacter lari* | 2.14 |
| AGR5017 | Site 3 | 5 | 1/09/2020 | *Campylobacter lari* | 2.17 |
| AGR5153 | Site 2 | 6 | 1/10/2020 | *Campylobacter coli* | 2.16 |
| AGR5154 | Site 2 | 6 | 1/10/2020 | *Campylobacter coli* | 2.18 |
| AGR5155 | Site 2 | 6 | 1/10/2020 | *Campylobacter coli* | 2.16 |
| AGR5156 | Site 3 | 6 | 1/10/2020 | NOIP | 1.54 |
| AGR5157 | Site 3 | 6 | 1/10/2020 | NOIP | 1.52 |
| AGR5232 | Site 1 | 7 | 2/11/2020 | *Campylobacter jejuni* | 2.53 |
| AGR5233 | Site 1 | 7 | 2/11/2020 | *Campylobacter jejuni* | 2.39 |
| AGR5235 | Site 2 | 7 | 2/11/2020 | *Campylobacter coli* | 2.14 |
| AGR5236 | Site 2 | 7 | 2/11/2020 | *Campylobacter coli* | 2.12 |
| AGR5237 | Site 3 | 7 | 2/11/2020 | *Campylobacter coli* | 2.10 |
| AGR5238 | Site 3 | 7 | 2/11/2020 | *Campylobacter coli* | 1.90 |
| AGR5876 | Site 1 | 8 | 14/12/2020 | NOIP | 1.44 |
| AGR5877 | Site 1 | 8 | 14/12/2020 | *Campylobacter jejuni* | 2.30 |
| AGR5878 | Site 2 | 8 | 14/12/2020 | *Campylobacter jejuni* | 2.24 |
| AGR5879 | Site 2 | 8 | 14/12/2020 | *Campylobacter jejuni* | 2.29 |
| AGR5880 | Site 3 | 8 | 14/12/2020 | *Campylobacter jejuni* | 2.17 |
| AGR5881 | Site 3 | 8 | 14/12/2020 | *Campylobacter jejuni* | 2.37 |
| AGR5916 | Site 1 | 9 | 21/01/2021 | *Campylobacter jejuni* | 2.33 |
| AGR5917 | Site 1 | 9 | 21/01/2021 | *Campylobacter jejuni* | 2.31 |
| AGR5918 | Site 1 | 9 | 21/01/2021 | *Campylobacter jejuni* | 2.27 |
| AGR5919 | Site 2 | 9 | 21/01/2021 | *Campylobacter coli* | 2.11 |
| AGR5920 | Site 2 | 9 | 21/01/2021 | *Campylobacter coli* | 2.08 |
| AGR5921 | Site 2 | 9 | 21/01/2021 | *Campylobacter coli* | 2.13 |
| AGR5922 | Site 2 | 9 | 21/01/2021 | *Campylobacter coli* | 2.22 |
| AGR5923 | Site 2 | 9 | 21/01/2021 | *Campylobacter coli* | 2.08 |
| AGR5924 | Site 2 | 9 | 21/01/2021 | *Campylobacter coli* | 2.14 |
| AGR5925 | Site 3 | 9 | 21/01/2021 | *Campylobacter jejuni* | 2.30 |
| AGR5926 | Site 3 | 9 | 21/01/2021 | *Campylobacter jejuni* | 2.30 |
| AGR5927 | Site 3 | 9 | 21/01/2021 | *Campylobacter jejuni* | 2.25 |
| AGR6130 | Site 2 | 10 | 23/02/2021 | *Campylobacter jejuni* | 2.31 |
| AGR6131 | Site 2 | 10 | 23/02/2021 | NOIP | 1.68 |
| AGR6132 | Site 2 | 10 | 23/02/2021 | *Campylobacter jejuni* | 2.31 |
| AGR6133 | Site 2 | 10 | 23/02/2021 | *Campylobacter jejuni* | 2.16 |
| AGR6134 | Site 3 | 10 | 23/02/2021 | *Campylobacter jejuni* | 2.32 |
| AGR6135 | Site 3 | 10 | 23/02/2021 | *Campylobacter jejuni* | 2.36 |
| AGR6136 | Site 3 | 10 | 23/02/2021 | *Campylobacter jejuni* | 2.23 |
| AGR6139 | Site 2 | 11 | 9/03/2021 | *Campylobacter jejuni* | 2.17 |
| AGR6140 | Site 2 | 11 | 9/03/2021 | *Campylobacter jejuni* | 2.16 |
| AGR6141 | Site 2 | 11 | 9/03/2021 | *Campylobacter jejuni* | 2.33 |
| AGR6142 | Site 3 | 11 | 9/03/2021 | *Campylobacter jejuni* | 2.44 |
| AGR6143 | Site 3 | 11 | 9/03/2021 | NOIP | 1.42 |
| AGR6144 | Site 3 | 11 | 9/03/2021 | *Campylobacter jejuni* | 2.42 |
| AGR6145 | Site 3 | 11 | 9/03/2021 | *Campylobacter jejuni* | 2.42 |
| AGR6146 | Site 3 | 11 | 9/03/2021 | *Campylobacter jejuni* | 2.43 |

***Table S3.*** Whole genome sequencing assembly details and identification of *Campylobacter* (n=84).

|  |  |  |  |  |  |  |  | ***Centrifuge ids*** | | | | | |
| --- | --- | --- | --- | --- | --- | --- | --- | --- | --- | --- | --- | --- | --- |
| ***Isolate*** | ***Contigs*** | ***bp*** | ***avg*** | ***max*** | ***N50*** | ***CDS*** | ***Accession*** | ***#1 Match*** | ***%*** | ***#2 Match*** | ***%*** | ***#3 Match*** | ***%*** |
| AGR4590 | 40 | 1622897 | 40572 | 290824 | 100689 | 1580 | SAMN42652974 | *C. jejuni* | 80.6 | unclassified | 7.1 | *C. coli* | 5.9 |
| AGR4591 | 48 | 1617857 | 33705 | 290930 | 61620 | 1579 | SAMN42652975 | *C. jejuni* | 78.7 | unclassified | 8.2 | *C. coli* | 6.0 |
| AGR4593 | 62 | 1914927 | 30885 | 202947 | 79794 | 1917 | SAMN42652976 | unclassified | 34.3 | *C. coli* | 22.5 | *C. jejuni* | 20.5 |
| AGR4594 | 46 | 1594558 | 34664 | 383995 | 78669 | 1550 | SAMN42652977 | *C. jejuni* | 80.0 | unclassified | 9.5 | *C. coli* | 5.1 |
| AGR4595 | 60 | 1591947 | 26532 | 162476 | 61382 | 1547 | SAMN42652978 | *C. jejuni* | 80.8 | unclassified | 8.6 | *C. coli* | 5.1 |
| AGR4596 | 72 | 1910703 | 26537 | 200323 | 75027 | 1913 | SAMN42652979 | unclassified | 32.0 | *C. coli* | 22.5 | *C. jejuni* | 21.7 |
| AGR4597 | 66 | 1816458 | 27522 | 206114 | 129781 | 1855 | SAMN42652980 | *C. coli* | 80.8 | *C. jejuni* | 7.6 | unclassified | 5.0 |
| AGR4598 | 69 | 1814738 | 26300 | 206046 | 129027 | 1852 | SAMN42652981 | *C. coli* | 81.6 | *C. jejuni* | 7.2 | unclassified | 4.8 |
| AGR4599 | 61 | 1855480 | 30417 | 181068 | 140967 | 1918 | SAMN42652982 | *C. coli* | 78.8 | *C. jejuni* | 8.4 | unclassified | 5.7 |
| AGR4600 | 82 | 1849234 | 22551 | 199165 | 127884 | 1903 | SAMN42652983 | *C. coli* | 79.0 | *C. jejuni* | 8.2 | unclassified | 5.4 |
| AGR4601 | 69 | 1851293 | 26830 | 260523 | 160414 | 1909 | SAMN42652984 | *C. coli* | 77.3 | *C. jejuni* | 8.5 | unclassified | 6.4 |
| AGR4602 | 69 | 1851606 | 26834 | 207812 | 140968 | 1910 | SAMN42652985 | *C. coli* | 78.4 | *C. jejuni* | 7.8 | unclassified | 5.9 |
| AGR4604 | 56 | 1912196 | 34146 | 265610 | 73206 | 1918 | SAMN42652986 | unclassified | 34.5 | *C. coli* | 21.6 | *C. jejuni* | 20.9 |
| AGR4605 | 65 | 1907382 | 29344 | 265607 | 68452 | 1912 | SAMN42652987 | unclassified | 33.4 | *C. coli* | 22.4 | *C. jejuni* | 20.7 |
| AGR4682 | 33 | 1713466 | 51923 | 268416 | 136602 | 1722 | SAMN42652988 | *C. coli* | 87.0 | *C. jejuni* | 4.3 | unclassified | 4.1 |
| AGR4683 | 36 | 1713396 | 47594 | 201566 | 119063 | 1723 | SAMN42652989 | *C. coli* | 88.6 | *C. jejuni* | 3.7 | unclassified | 3.7 |
| AGR4685 | 36 | 1720214 | 47783 | 228112 | 114572 | 1727 | SAMN42652990 | *C. coli* | 86.8 | unclassified | 5.0 | *C. jejuni* | 4.0 |
| AGR4687 | 35 | 1720968 | 49170 | 229519 | 100293 | 1731 | SAMN42652991 | *C. coli* | 83.6 | unclassified | 8.5 | *C. jejuni* | 3.5 |
| AGR4688 | 98 | 1811422 | 18483 | 123130 | 68593 | 1814 | SAMN42652992 | *C. coli* | 80.2 | *C. jejuni* | 6.7 | unclassified | 6.5 |
| AGR4689 | 15 | 1562848 | 104189 | 272110 | 173430 | 1583 | SAMN42652993 | *C. jejuni* | 88.1 | *C. coli* | 3.4 | unclassified | 1.0 |
| AGR4691 | 29 | 1675794 | 57786 | 198101 | 144340 | 1666 | SAMN42652994 | *C. coli* | 87.4 | *C. jejuni* | 4.4 | unclassified | 3.8 |
| AGR4732 | 28 | 1676243 | 59865 | 198053 | 143597 | 1666 | SAMN42652995 | *C. coli* | 86.8 | *C. jejuni* | 4.8 | unclassified | 4.0 |
| AGR4733 | 60 | 1670274 | 27837 | 118226 | 79801 | 1656 | SAMN42652996 | *C. coli* | 86.7 | *C. jejuni* | 5.5 | unclassified | 3.7 |
| AGR4734 | 38 | 1718607 | 45226 | 198637 | 109567 | 1725 | SAMN42652997 | *C. coli* | 87.7 | unclassified | 4.5 | *C. jejuni* | 3.8 |
| AGR4735 | 34 | 1720055 | 50589 | 198958 | 113704 | 1725 | SAMN42652998 | *C. coli* | 86.2 | unclassified | 5.5 | *C. jejuni* | 3.7 |
| AGR4736 | 35 | 1713750 | 48964 | 207318 | 113973 | 1716 | SAMN42652999 | *C. coli* | 88.0 | unclassified | 3.8 | *C. jejuni* | 3.8 |
| AGR4737 | 36 | 1713770 | 47604 | 263577 | 151406 | 1722 | SAMN42653000 | *C. coli* | 86.1 | *C. jejuni* | 5.7 | unclassified | 3.7 |
| AGR4738 | 35 | 1715517 | 49014 | 198592 | 120018 | 1727 | SAMN42653001 | *C. coli* | 85.5 | *C. jejuni* | 6.2 | unclassified | 3.9 |
| AGR4739 | 46 | 1715590 | 37295 | 198101 | 120014 | 1725 | SAMN42653002 | *C. coli* | 86.1 | *C. jejuni* | 5.3 | unclassified | 3.8 |
| AGR4960 | 85 | 1746748 | 20549 | 316818 | 138848 | 1746 | SAMN42653003 | *C. jejuni* | 85.7 | *C. coli* | 4.7 | unclassified | 2.2 |
| AGR4962 | 22 | 1601461 | 72793 | 282367 | 150200 | 1662 | SAMN42653004 | *C. jejuni* | 87.1 | *C. coli* | 4.5 | unclassified | 0.7 |
| AGR4963 | 37 | 1597312 | 43170 | 315457 | 85763 | 1663 | SAMN42653005 | *C. jejuni* | 86.7 | *C. coli* | 4.5 | unclassified | 0.6 |
| AGR4964 | 29 | 1720543 | 59329 | 229410 | 114573 | 1734 | SAMN42653006 | *C. coli* | 87.2 | unclassified | 4.7 | *C. jejuni* | 3.9 |
| AGR4965 | 70 | 1793296 | 25618 | 139422 | 67018 | 1824 | SAMN42653007 | *C. coli* | 87.7 | *C. jejuni* | 4.3 | unclassified | 3.9 |
| AGR4966 | 43 | 1685840 | 39205 | 475673 | 100920 | 1725 | SAMN42653008 | *C. jejuni* | 85.8 | *C. coli* | 5.1 | unclassified | 0.5 |
| AGR4967 | 57 | 1821050 | 31948 | 231413 | 160887 | 1861 | SAMN42653009 | *C. coli* | 80.4 | *C. jejuni* | 8.0 | unclassified | 5.0 |
| AGR4969 | 61 | 1817542 | 29795 | 181373 | 129030 | 1859 | SAMN42653010 | *C. coli* | 79.9 | *C. jejuni* | 7.7 | unclassified | 5.3 |
| AGR5006 | 23 | 1709079 | 74307 | 228112 | 130968 | 1718 | SAMN42653011 | *C. coli* | 88.3 | *C. jejuni* | 3.9 | unclassified | 3.7 |
| AGR5007 | 30 | 1708531 | 56951 | 228112 | 103757 | 1715 | SAMN42653012 | *C. coli* | 87.9 | *C. jejuni* | 3.9 | unclassified | 3.7 |
| AGR5008 | 86 | 1699716 | 19764 | 111123 | 40463 | 1705 | SAMN42653013 | *C. coli* | 88.6 | unclassified | 3.7 | *C. jejuni* | 3.6 |
| AGR5009 | 79 | 1712847 | 21681 | 142121 | 73055 | 1696 | SAMN42653014 | unclassified | 48.8 | *C. jejuni* | 14.6 | *C. coli* | 12.3 |
| AGR5010 | 83 | 1699164 | 20471 | 198217 | 47642 | 1687 | SAMN42653015 | *C. coli* | 88.2 | unclassified | 3.7 | *C. jejuni* | 3.6 |
| AGR5011 | 109 | 1691025 | 15513 | 107138 | 33773 | 1684 | SAMN42653016 | *C. coli* | 88.6 | unclassified | 3.7 | *C. jejuni* | 3.7 |
| AGR5012 | 91 | 1695050 | 18626 | 128810 | 46026 | 1693 | SAMN42653017 | *C. coli* | 88.4 | unclassified | 3.8 | *C. jejuni* | 3.5 |
| AGR5013 | 58 | 1702104 | 29346 | 162463 | 69803 | 1705 | SAMN42653018 | *C. coli* | 88.0 | unclassified | 3.9 | *C. jejuni* | 3.7 |
| AGR5014 | 34 | 1485471 | 43690 | 199951 | 92050 | 1511 | SAMN42653019 | *C. lari* | 93.9 | *C. subantarcticus* | 1.3 | unclassified | 1.2 |
| AGR5017 | 27 | 1451160 | 53746 | 295789 | 145089 | 1465 | SAMN42653020 | *C. lari* | 93.2 | *C. subantarcticus* | 1.3 | *C. sp. RM16704* | 0.9 |
| AGR5153 | 53 | 1708757 | 32240 | 168733 | 79763 | 1711 | SAMN42653021 | *C. coli* | 87.5 | unclassified | 5.0 | *C. jejuni* | 3.4 |
| AGR5155 | 75 | 1704588 | 22727 | 128491 | 55723 | 1704 | SAMN42653022 | *C. coli* | 86.8 | unclassified | 5.2 | *C. jejuni* | 3.7 |
| AGR5156 | 93 | 2010960 | 21623 | 239407 | 54176 | 2017 | SAMN42653023 | unclassified | 34.0 | *C. coli* | 21.9 | *C. jejuni* | 20.6 |
| AGR5157 | 101 | 2012187 | 19922 | 217075 | 68382 | 2011 | SAMN42653024 | unclassified | 36.8 | *C. coli* | 21.9 | *C. jejuni* | 18.8 |
| AGR5232 | 30 | 1678787 | 55959 | 286911 | 117300 | 1745 | SAMN42653025 | *C. jejuni* | 86.1 | *C. coli* | 4.4 | unclassified | 0.8 |
| AGR5233 | 35 | 1677133 | 47918 | 286868 | 123684 | 1745 | SAMN42653026 | *C. jejuni* | 84.8 | *C. coli* | 4.3 | unclassified | 1.1 |
| AGR5235 | 48 | 1719496 | 35822 | 219826 | 80160 | 1727 | SAMN42653027 | *C. coli* | 85.5 | *C. jejuni* | 5.3 | unclassified | 5.3 |
| AGR5236 | 61 | 1718558 | 28173 | 131419 | 68935 | 1727 | SAMN42653028 | *C. coli* | 84.4 | unclassified | 5.8 | *C. jejuni* | 5.2 |
| AGR5237 | 63 | 1818301 | 28861 | 231411 | 160952 | 1864 | SAMN42653029 | *C. coli* | 80.2 | *C. jejuni* | 7.7 | unclassified | 5.4 |
| AGR5238 | 64 | 1818120 | 28408 | 175280 | 130921 | 1861 | SAMN42653030 | *C. coli* | 81.1 | *C. jejuni* | 7.2 | unclassified | 4.7 |
| AGR5876 | 119 | 2052201 | 17245 | 223529 | 42121 | 2056 | SAMN42653031 | unclassified | 34.2 | *C. coli* | 23.0 | *C. jejuni* | 19.9 |
| AGR5877 | 73 | 1707333 | 23388 | 146153 | 66038 | 1719 | SAMN42653032 | *C. jejuni* | 89.7 | *C. coli* | 3.3 | unclassified | 1.8 |
| AGR5878 | 27 | 1728279 | 64010 | 285499 | 150326 | 1808 | SAMN42653033 | *C. jejuni* | 84.8 | *C. coli* | 5.2 | unclassified | 0.8 |
| AGR5879 | 46 | 1648231 | 35831 | 187740 | 84279 | 1664 | SAMN42653034 | *C. jejuni* | 92.1 | *C. coli* | 1.8 | unclassified | 0.5 |
| AGR5880 | 40 | 1649409 | 41235 | 166796 | 77715 | 1672 | SAMN42653035 | *C. jejuni* | 91.6 | *C. coli* | 1.8 | unclassified | 0.6 |
| AGR5881 | 41 | 1646262 | 40152 | 148061 | 73787 | 1674 | SAMN42653036 | *C. jejuni* | 92.1 | *C. coli* | 1.8 | unclassified | 0.5 |
| AGR5916 | 34 | 1593990 | 46882 | 185461 | 97932 | 1595 | SAMN42653037 | *C. jejuni* | 92.8 | *C. coli* | 2.0 | unclassified | 0.4 |
| AGR5917 | 30 | 1604020 | 53467 | 227701 | 134797 | 1614 | SAMN42653038 | *C. jejuni* | 92.9 | *C. coli* | 1.6 | unclassified | 0.5 |
| AGR5918 | 34 | 1595374 | 46922 | 184885 | 86900 | 1594 | SAMN42653039 | *C. jejuni* | 92.1 | *C. coli* | 2.0 | unclassified | 0.5 |
| AGR5919 | 57 | 1716767 | 30118 | 157157 | 77153 | 1717 | SAMN42653040 | *C. coli* | 86.8 | unclassified | 5.3 | *C. jejuni* | 3.7 |
| AGR5921 | 56 | 1718451 | 30686 | 168659 | 65457 | 1722 | SAMN42653041 | *C. coli* | 86.9 | unclassified | 5.2 | *C. jejuni* | 3.7 |
| AGR5922 | 51 | 1717432 | 33675 | 168659 | 78657 | 1722 | SAMN42653042 | *C. coli* | 85.1 | unclassified | 7.1 | *C. jejuni* | 3.7 |
| AGR5923 | 33 | 1718287 | 52069 | 228115 | 108441 | 1731 | SAMN42653043 | *C. coli* | 86.8 | unclassified | 5.1 | *C. jejuni* | 3.8 |
| AGR5925 | 52 | 1603823 | 30842 | 158759 | 73281 | 1617 | SAMN42653044 | *C. jejuni* | 92.4 | *C. coli* | 2.1 | unclassified | 0.5 |
| AGR6130 | 59 | 1641497 | 27821 | 135913 | 53327 | 1706 | SAMN42653045 | *C. jejuni* | 95.8 | *C. coli* | 0.6 | unclassified | 0.6 |
| AGR6131 | 93 | 2002092 | 21527 | 195149 | 53225 | 2001 | SAMN42653046 | unclassified | 32.3 | *C. coli* | 21.5 | *C. jejuni* | 21.2 |
| AGR6132 | 44 | 1591183 | 36163 | 191352 | 75867 | 1583 | SAMN42653047 | *C. jejuni* | 91.8 | *C. coli* | 2.5 | unclassified | 1.6 |
| AGR6133 | 42 | 1648346 | 39246 | 295123 | 120276 | 1622 | SAMN42653048 | *C. jejuni* | 82.5 | unclassified | 6.8 | *C. coli* | 5.2 |
| AGR6134 | 21 | 1543328 | 73491 | 364468 | 155310 | 1600 | SAMN42653049 | *C. jejuni* | 86.7 | *C. coli* | 4.4 | *unclassified* | 2.9 |
| AGR6135 | 28 | 1646377 | 58799 | 257351 | 134018 | 1718 | SAMN42653050 | *C. jejuni* | 91.6 | unclassified | 1.9 | *C. coli* | 0.7 |
| AGR6136 | 33 | 1646522 | 49894 | 145155 | 105440 | 1716 | SAMN42653051 | *C. jejuni* | 94.6 | unclassified | 0.9 | *C. coli* | 0.6 |
| AGR6139 | 57 | 1593528 | 27956 | 293430 | 63350 | 1550 | SAMN42653052 | *C. jejuni* | 81.7 | unclassified | 7.8 | *C. coli* | 5.1 |
| AGR6141 | 51 | 1718001 | 33686 | 315277 | 145452 | 1731 | SAMN42653053 | *C. jejuni* | 89.3 | *C. coli* | 3.9 | unclassified | 1.9 |
| AGR6142 | 20 | 1592975 | 79648 | 221310 | 152085 | 1586 | SAMN42653054 | *C. jejuni* | 92.3 | *C. coli* | 2.3 | unclassified | 1.4 |
| AGR6143 | 72 | 2015080 | 27987 | 215961 | 74535 | 2016 | SAMN42653055 | unclassified | 32.7 | *C. coli* | 21.5 | *C. jejuni* | 20.9 |
| AGR6144 | 20 | 1593997 | 79699 | 221310 | 151521 | 1589 | SAMN42653056 | *C. jejuni* | 92.4 | *C. coli* | 2.3 | unclassified | 1.4 |
| AGR6146 | 25 | 1592565 | 63702 | 186870 | 138242 | 1585 | SAMN42653057 | *C. jejuni* | 92.4 | *C. coli* | 2.3 | unclassified | 1.4 |

***Table S4.*** MLST sequence types and antimicrobial resistance genes identified from Campylobacter (n=84) whole genome sequence data. * UA - unassigned using *C. coli*/*C. jejuni* MLST scheme, # bla_OXA_ from AGR5009 was < 80% similar to bla_OXA-616_ at the amino acid sequence level.

| **Isolate** | **ID/ST** | **ARGs** |
| --- | --- | --- |
| AGR4590 | 11284 | bla_OXA-620_ |
| AGR4591 | 11284 | bla_OXA-620_ |
| AGR4593 | UA* | bla_OXA-583_ |
| AGR4594 | 2381 | bla_OXA-620_ |
| AGR4595 | 2381 | bla_OXA-620_ |
| AGR4596 | UA | bla_OXA-583_ |
| AGR4597 | 7774 | aadE-Cc |
| AGR4598 | 7774 | aadE-Cc |
| AGR4599 | 7774 | aadE-Cc |
| AGR4600 | 7774 | aadE-Cc |
| AGR4601 | 7774 | aadE-Cc |
| AGR4602 | 7774 | aadE-Cc |
| AGR4604 | UA | bla_OXA-583_ |
| AGR4605 | UA | bla_OXA-583_ |
| AGR4682 | 3302 | aadE-Cc |
| AGR4683 | 3302 | aadE-Cc |
| AGR4685 | 3302 | aadE-Cc |
| AGR4687 | 3302 | aadE-Cc |
| AGR4688 | 8718 | none |
| AGR4689 | 1276 | bla_OXA-631_ |
| AGR4691 | 3302 | aadE-Cc |
| AGR4732 | 3302 | aadE-Cc |
| AGR4733 | 3302 | aadE-Cc |
| AGR4734 | 3302 | aadE-Cc |
| AGR4735 | 3302 | aadE-Cc |
| AGR4736 | 3302 | aadE-Cc |
| AGR4737 | 3302 | aadE-Cc |
| AGR4738 | 3302 | aadE-Cc |
| AGR4739 | 3302 | aadE-Cc |
| AGR4960 | 14290 | none |
| AGR4962 | 1956 | bla_OXA-184_ |
| AGR4963 | 1956 | bla_OXA-184_ |
| AGR4964 | 3302 | aadE-Cc |
| AGR4965 | 3302 | aadE-Cc |
| AGR4966 | 2391 | bla_OXA-466_ |
| AGR4967 | 7774 | aadE-Cc |
| AGR4969 | 7774 | aadE-Cc |
| AGR5006 | 3302 | aadE-Cc |
| AGR5007 | 3302 | aadE-Cc |
| AGR5008 | 3302 | aadE-Cc |
| AGR5009 | UA | ^#^bla_OXA-616_ |
| AGR5010 | 3302 | aadE-Cc |
| AGR5011 | 3302 | aadE-Cc |
| AGR5012 | 3302 | aadE-Cc |
| AGR5013 | 3302 | aadE-Cc |
| AGR5014 | *C. lari* | bla_OXA-493_ |
| AGR5017 | *C. lari* | bla_OXA-493_ |
| AGR5153 | 3302 | aadE-Cc |
| AGR5155 | 3302 | aadE-Cc |
| AGR5156 | UA | bla_OXA-583_ |
| AGR5157 | UA | bla_OXA-583_ |
| AGR5232 | 257 | bla_OXA-605_ |
| AGR5233 | 257 | bla_OXA-605_ |
| AGR5235 | 3302 | aadE-Cc |
| AGR5236 | 3302 | aadE-Cc |
| AGR5237 | 7774 | aadE-Cc |
| AGR5238 | 7774 | aadE-Cc |
| AGR5876 | UA | bla_OXA-583_ |
| AGR5877 | 14291 | none |
| AGR5878 | 61 | bla_OXA-605_ |
| AGR5879 | 45 | bla_OXA-605_ |
| AGR5880 | 45 | bla_OXA-605_ |
| AGR5881 | 45 | bla_OXA-605_ |
| AGR5916 | 45 | bla_OXA-605_ |
| AGR5917 | 45 | bla_OXA-184_ |
| AGR5918 | 45 | bla_OXA-605_ |
| AGR5919 | 3302 | aadE-Cc |
| AGR5921 | 3302 | aadE-Cc |
| AGR5922 | 3302 | aadE-Cc |
| AGR5923 | 3302 | aadE-Cc |
| AGR5925 | 45 | bla_OXA-447_ |
| AGR6130 | 677 | bla_OXA-447_ |
| AGR6131 | UA | bla_OXA-583_ |
| AGR6132 | 177 | bla_OXA-447_ |
| AGR6133 | 2381 | bla_OXA-620_ |
| AGR6134 | 1286 | bla_OXA-616_ |
| AGR6135 | 677 | bla_OXA-447_ |
| AGR6136 | 677 | bla_OXA-447_ |
| AGR6139 | 2381 | bla_OXA-620_ |
| AGR6141 | 14291 | none |
| AGR6142 | 177 | bla_OXA-447_, tetO |
| AGR6143 | UA | bla_OXA-583_ |
| AGR6144 | 177 | bla_OXA-447_, tetO |
| AGR6146 | 177 | bla_OXA-447_, tetO |

***Table S5.*** Antibiotic sensitivity testing zone sizes (mm). * Not done.

| **Isolate ID** | **Nalidixic Acid** | **Ampicillin** | **Gentamicin** | **PenicillinG** | **Ciprofloxacin** | **Erythromycin** | **Tetracycline** |
| --- | --- | --- | --- | --- | --- | --- | --- |
| AGR4590 | 29 | 32 | 32 | 6 | 45 | 37 | 41 |
| AGR4591 | 27 | 31 | 28 | 6 | 42 | 33 | 41 |
| AGR4593 | 29 | 34 | 27 | 6 | 41 | 36 | 43 |
| AGR4594 | 25 | 30 | 32 | 6 | 42 | 32 | 38 |
| AGR4595 | 33 | 33 | 31 | 6 | 41 | 34 | 44 |
| AGR4596 | 27 | 15 | 26 | 6 | 40 | 32 | 41 |
| AGR4597 | 30 | 36 | 31 | 6 | 47 | 39 | 43 |
| AGR4598 | 34 | 33 | 32 | 6 | 47 | 41 | 48 |
| AGR4599 | 26 | 30 | 32 | 6 | 42 | 34 | 39 |
| AGR4600 | 33 | 32 | 31 | 6 | 45 | 37 | 45 |
| AGR4601 | 28 | 34 | 32 | 6 | 44 | 38 | 42 |
| AGR4602 | 31 | 32 | 31 | 6 | 44 | 38 | 45 |
| AGR4603 | 23 | 18 | 25 | 6 | 36 | 26 | 43 |
| AGR4604 | 27 | 32 | 31 | 6 | 40 | 34 | 39 |
| AGR4604 | 25 | 25 | 27 | 6 | 35 | 31 | 42 |
| AGR4605 | 27 | 25 | 25 | 6 | 38 | 32 | 40 |
| AGR4682 | 28 | 30 | 29 | 6 | 43 | 33 | 39 |
| AGR4683 | 34 | 33 | 34 | 6 | 49 | 41 | 49 |
| AGR4684 | 39 | 31 | 30 | 6 | 43 | 36 | 38 |
| AGR4685 | 30 | 36 | 29 | 6 | 41 | 34 | 41 |
| AGR4686 | *ND | ND | ND | ND | ND | ND | ND |
| AGR4687 | 29 | 32 | 32 | 6 | 44 | 36 | 41 |
| AGR4688 | 23 | 21 | 27 | 6 | 40 | 29 | 38 |
| AGR4689 | 25 | 21 | 26 | 6 | 36 | 30 | 37 |
| AGR4690 | 28 | 16 | 30 | 6 | 34 | 34 | 44 |
| AGR4691 | 29 | 35 | 29 | 17 | 43 | 35 | 43 |
| AGR4732 | 25 | 23 | 25 | 6 | 40 | 33 | 41 |
| AGR4733 | 29 | 34 | 29 | 6 | 44 | 36 | 44 |
| AGR4734 | 26 | 27 | 30 | 6 | 40 | 33 | 37 |
| AGR4735 | 25 | 24 | 27 | 6 | 39 | 32 | 39 |
| AGR4736 | 28 | 32 | 31 | 6 | 43 | 34 | 43 |
| AGR4737 | 30 | 31 | 32 | 6 | 43 | 37 | 44 |
| AGR4738 | 27 | 33 | 29 | 6 | 44 | 35 | 41 |
| AGR4739 | 32 | 34 | 32 | 6 | 40 | 36 | 44 |
| AGR4740 | 28 | 31 | 30 | 6 | 41 | 35 | 40 |
| AGR4960 | 27 | 28 | 29 | 6 | 35 | 29 | 43 |
| AGR4961 | 26 | 6 | 27 | 6 | 35 | 30 | 37 |
| AGR4962 | 29 | 6 | 29 | 6 | 38 | 30 | 38 |
| AGR4963 | 26 | 6 | 27 | 6 | 38 | 31 | 39 |
| AGR4964 | 30 | 32 | 30 | 6 | 40 | 34 | 43 |
| AGR4965 | 27 | 29 | 34 | 6 | 47 | 40 | 46 |
| AGR4966 | 28 | 27 | 28 | 6 | 39 | 31 | 44 |
| AGR4967 | 33 | 35 | 34 | 6 | 42 | 41 | 44 |
| AGR4968 | 30 | 29 | 31 | 6 | 42 | 36 | 43 |
| AGR4969 | 31 | 31 | 31 | 6 | 46 | 40 | 48 |
| AGR5006 | 27 | 31 | 28 | 6 | 41 | 36 | 42 |
| AGR5007 | 28 | 30 | 32 | 6 | 45 | 36 | 41 |
| AGR5008 | 28 | 32 | 32 | 6 | 44 | 36 | 44 |
| AGR5009 | 25 | 23 | 25 | 6 | 36 | 28 | 40 |
| AGR5010 | 30 | 31 | 31 | 6 | 40 | 34 | 45 |
| AGR5011 | 29 | 29 | 30 | 6 | 44 | 37 | 43 |
| AGR5012 | 28 | 30 | 30 | 6 | 47 | 38 | 41 |
| AGR5013 | 27 | 25 | 27 | 6 | 37 | 30 | 42 |
| AGR5014 | 28 | 30 | 30 | 6 | 46 | 35 | 43 |
| AGR5015 | 24 | 21 | 26 | 11 | 34 | 26 | 39 |
| AGR5016 | 25 | 6 | 26 | 6 | 32 | 30 | 42 |
| AGR5017 | 21 | 6 | 26 | 6 | 34 | 30 | 38 |
| AGR5153 | 29 | 29 | 30 | 6 | 41 | 30 | 45 |
| AGR5154 | 27 | 31 | 31 | 6 | 41 | 35 | 41 |
| AGR5155 | 28 | 30 | 29 | 6 | 43 | 35 | 40 |
| AGR5156 | 28 | 28 | 29 | 6 | 39 | 33 | 38 |
| AGR5157 | 28 | 18 | 28 | 6 | 40 | 32 | 42 |
| AGR5232 | 27 | 29 | 27 | 6 | 40 | 35 | 41 |
| AGR5233 | 28 | 17 | 26 | 6 | 38 | 32 | 40 |
| AGR5235 | 29 | 33 | 31 | 6 | 41 | 35 | 40 |
| AGR5236 | 27 | 29 | 30 | 6 | 40 | 33 | 42 |
| AGR5237 | 27 | 30 | 30 | 6 | 42 | 34 | 40 |
| AGR5238 | 29 | 33 | 29 | 6 | 43 | 35 | 40 |
| AGR5876 | 27 | 29 | 32 | 6 | 44 | 36 | 40 |
| AGR5877 | 26 | 27 | 30 | 6 | 38 | 31 | 40 |
| AGR5878 | 27 | 30 | 32 | 6 | 43 | 35 | 41 |
| AGR5879 | 29 | 25 | 29 | 6 | 40 | 33 | 45 |
| AGR5880 | 28 | 25 | 28 | 6 | 40 | 32 | 42 |
| AGR5881 | 28 | 26 | 27 | 6 | 40 | 31 | 43 |
| AGR5916 | 29 | 30 | 30 | 6 | 42 | 38 | 43 |
| AGR5917 | 31 | 10 | 27 | 6 | 42 | 33 | 44 |
| AGR5918 | 29 | 25 | 29 | 6 | 41 | 34 | 44 |
| AGR5919 | 27 | 24 | 26 | 6 | 36 | 29 | 41 |
| AGR5920 | 29 | 28 | 30 | 6 | 39 | 35 | 39 |
| AGR5921 | 29 | 26 | 31 | 6 | 44 | 34 | 42 |
| AGR5922 | 28 | 27 | 30 | 6 | 43 | 37 | 41 |
| AGR5923 | 29 | 27 | 30 | 6 | 46 | 36 | 41 |
| AGR5924 | 29 | 26 | 30 | 6 | 39 | 36 | 40 |
| AGR5925 | 25 | 24 | 25 | 6 | 42 | 33 | 42 |
| AGR5926 | 31 | 25 | 30 | 6 | 38 | 33 | 44 |
| AGR5927 | 27 | 23 | 29 | 6 | 40 | 34 | 42 |
| AGR6130 | 26 | 21 | 27 | 6 | 39 | 34 | 40 |
| AGR6131 | 30 | 27 | 30 | 6 | 42 | 33 | 45 |
| AGR6132 | 23 | 19 | 26 | 6 | 35 | 24 | 42 |
| AGR6133 | 30 | 29 | 31 | 6 | 43 | 33 | 42 |
| AGR6134 | 21 | 14 | 33 | 6 | 36 | 34 | 46 |
| AGR6135 | 26 | 20 | 27 | 6 | 40 | 30 | 40 |
| AGR6136 | 25 | 19 | 25 | 6 | 41 | 28 | 39 |
| AGR6139 | 30 | 26 | 29 | 6 | 41 | 32 | 42 |
| AGR6140 | 36 | 28 | 32 | 6 | 36 | 36 | 44 |
| AGR6141 | 30 | 35 | 29 | 6 | 44 | 35 | 43 |
| AGR6142 | 30 | 20 | 28 | 6 | 43 | 32 | 6 |
| AGR6143 | 30 | 21 | 29 | 6 | 39 | 32 | 42 |
| AGR6144 | 23 | 20 | 27 | 6 | 36 | 27 | 6 |
| AGR6145 | 30 | 27 | 31 | 6 | 37 | 32 | 6 |
| AGR6146 | 32 | 22 | 30 | 6 | 44 | 35 | 6 |
| *C. jejuni* ATCC33560 | 26 | 30 | 29 | 6 | 37 | 30 | 36 |

***Table S6.*** Source attribution and geographical distribution data associated with *Campylobacter* sequence types (ST) identified in this study. Data obtained from PubMLST (July 2024). ‘Human’ totals include human stool, human blood culture and human unspecified; ‘Cattle’ totals include cattle, beef offal or meat, cattle faeces and calf; ‘Sheep’ totals include sheep, lamb, lamb offal or meat and sheep faeces; ‘Chicken’ totals include chicken and chicken offal or meat.

|  | ***ST (n)*** | ***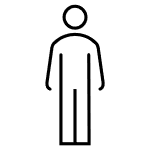*** | ***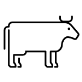*** | ***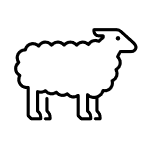*** | ***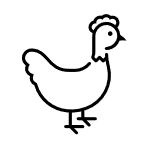*** | ***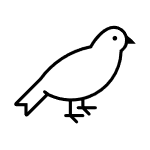*** | ***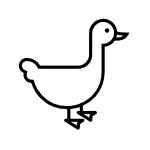*** | ***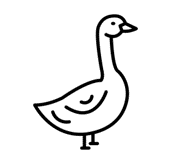*** | ***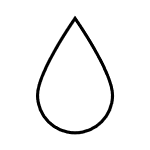*** | ***Unknown, other (n)*** | ***Countries of isolation*** |
| --- | --- | --- | --- | --- | --- | --- | --- | --- | --- | --- | --- |
| ***Campylobacter jejuni*** | ST-45 (4680) | 1873 | 259 | 29 | 1397 | 91 | 91 | 17 | 119 | 596, broiler env. (20), cat (12), carcass swab (4), cows milk (3), dog (26), ostrich (1), turkey (offal) (68), other animal (60) | Australia, Belgium, Brazil, Canada, China, Croatia, Curacao, Denmark, Egypt, Estonia, Finland, France, Germany, Greece, Italy, Japan, Lithuania, Lux, NZ, Norway, Peru, Slovenia, South Korea, Spain, Sweden, Switzerland, Thailand, Netherlands, Turkey, UK, USA |
|  | ST-61 (1969) | 747 | 652 | 215 | 72 | 1 | 0 | 0 | 24 | 228, cows milk (13), CSF, dog (2), giraffe, goat, turkey (6) | Australia, Bangladesh, Belgium, Canada, China, Estonia, Finland, France, Germany, Greece, Ireland, Italy, Japan, Lux, NZ, Norway, South Korea, Spain, Sweden, Switzerland, Netherlands, UK, USA |
|  | ST-177 (140) | 6 | 14 | 1 | 7 | 82 | 0 | 0 | 17 | 9, beach sand (3), other animal (1) | UK, Canada, Spain, Sweden, USA, Lux, NZ, France, Thailand, Italy, China |
|  | ST-257 (2800) | 2109 | 50 | 7 | 552 | 5 | 0 | 0 | 5 | 56, broiler env. (2), dog (4), turkey (7) | Australia, Austria, Bangladesh, Belgium, Canada, Chile, Croatia, Egypt, Estonia, France, Germany, Iran, Ireland, Israel, Italy, Japan, Lux, NZ, Norway, Poland, Slovenia, South Africa, Spain, Sweden, Switzerland, Netherlands, UK, USA |
|  | ST-677 (284) | 201 | 1 | 1 | 24 | 22 | 0 | 0 | 17 | 11, dog (4), human blood (8), other animal (2), rabbit (1) | Finland, France, Germany, Lux, NZ, Norway, Peru, Spain, Sweden, Switzerland, Netherlands, UK |
|  | ST-1276 (16) | 2 | 0 | 0 | 0 | 6 | 2 | 1 | 2 | 3 | Canada, Sweden, UK, USA, NZ |
|  | ST-1286 (18) | 2 | 0 | 0 | 0 | 9 | 0 | 0 | 7 | 0 | UK, Sweden, NZ, France |
|  | ST-1956 (15) | 6 | 0 | 0 | 2 | 1 | 6 | 0 | 0 | 0 | NZ, Finland, Canada, Lux, Switzerland, UK, USA |
|  | ST-2381 (37) | 0 | 0 | 0 | 0 | 2 | 0 | 0 | 35 | 0 | NZ |
|  | ST-2391 (9) | 0 | 0 | 0 | 2 | 4 | 2 | 0 | 1 | 0 | NZ |
|  | ST-11284 (4) | 0 | 0 | 0 | 0 | 0 | 0 | 0 | 4 | 0 | NZ |
| ***C. coli*** | ST-3302 (6) | 0 | 0 | 0 | 0 | 1 | 0 | 0 | 5 | 0 | NZ |
|  | ST-7774 (4) | 0 | 0 | 0 | 0 | 0 | 0 | 0 | 4 | 0 | NZ |
|  | ST-8178 (1) | 0 | 0 | 0 | 0 | 1 | 0 | 0 | 0 | 0 | NZ |
